# Tho2‐mediated escort of Nrd1 regulates the expression of aging‐related genes

**DOI:** 10.1111/acel.14203

**Published:** 2024-05-20

**Authors:** Yan Liu, Jeong‐Min Park, Suji Lim, Ruxin Duan, Do Yoon Lee, Dahee Choi, Dong Kyu Choi, Byung‐Ho Rhie, Soo Young Cho, Hong‐Yeoul Ryu, Seong Hoon Ahn

**Affiliations:** ^1^ Department of Molecular and Life Science, College of Science and Convergence Technology Hanyang University Ansan Republic of Korea; ^2^ KNU LAMP Research Center, KNU Institute of Basic Sciences, School of Life Sciences, BK21 FOUR KNU Creative BioResearch Group, College of Natural Sciences Kyungpook National University Daegu Republic of Korea; ^3^ School of Life Sciences, BK21 FOUR KNU Creative BioResearch Group, College of Natural Sciences Kyungpook National University Daegu Republic of Korea

**Keywords:** aging‐related genes, Nrd1, replicative lifespan, THO complex, transcription

## Abstract

The relationship between aging and RNA biogenesis and trafficking is attracting growing interest, yet the precise mechanisms are unknown. The THO complex is crucial for mRNA cotranscriptional maturation and export. Herein, we report that the THO complex is closely linked to the regulation of lifespan. Deficiencies in Hpr1 and Tho2, components of the THO complex, reduced replicative lifespan (RLS) and are linked to a novel Sir2‐independent RLS control pathway. Although transcript sequestration in *hpr1Δ* or *tho2Δ* mutants was countered by exosome component Rrp6, loss of this failed to mitigate RLS defects in *hpr1Δ*. However, RLS impairment in *hpr1Δ* or *tho2Δ* was counteracted by the additional expression of Nrd1‐specific mutants that interacted with Rrp6. This effect relied on the interaction of Nrd1, a transcriptional regulator of aging‐related genes, including ribosome biogenesis or RNA metabolism genes, with RNA polymerase II. Nrd1 overexpression reduced RLS in a Tho2‐dependent pathway. Intriguingly, *Tho2* deletion mirrored Nrd1 overexpression effects by inducing arbitrary Nrd1 chromatin binding. Furthermore, our genome‐wide ChIP‐seq analysis revealed an increase in the recruitment of Nrd1 to translation‐associated genes, known to be related to aging, upon Tho2 loss. Taken together, these findings underscore the importance of Tho2‐mediated Nrd1 escorting in the regulation of lifespan pathway through transcriptional regulation of aging‐related genes.

## Graphical abstract

Loss of Tho2 leads to an increase in Nrd1 recruitment to translation‐associated genes, known as aging‐related genes, thereby resulting in decreased expression of these genes. RLS impairment in tho2*Δ* is counteracted by the disturbance of WT Nrd1 function due to the presence of Nrd1 mutants.

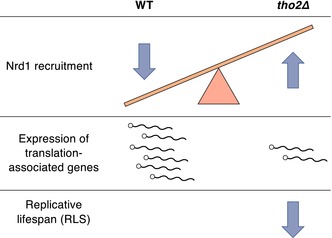

**Abbreviations**
CFU: colony forming unit
ChIP: chromatin immunoprecipitation
CID: CTD‐interaction domain
CLS: chronological lifespan
ERCs: extrachromosomal rDNA circles
GO: gene ontology
IGV: integrative genomics viewer
ncRNA: noncoding RNA
ORF: open reading frame
P/Q: proline‐ and glutamine‐rich region
PAR‐CLIP: photoactivatable ribonucleoside‐enhanced crosslinking and immunoprecipitation
RE/RS: arginine‐glutamine and arginine‐serine‐rich region
RLS: replicative lifespan
RRM: RNA recognition motif
SC: synthetic complete
TES: transcript end sites
TSS: transcription start site
UTR: untranslated region
YPD: yeast extract peptone dextrose

## 1 INTRODUCTION

Aging is characterized by the gradual accumulation of molecular, cellular, and organ damage that causes the decline of biological functions and heightened susceptibility to morbidity and mortality (Fontana et al, 2010; Jang et al., 2023; Lai et al., 2019; Whang et al., 2021). The budding yeast *Saccharomyces cerevisiae* is a valuable model in aging studies using replicative lifespan (RLS) and chronological lifespan (CLS) assays (Longo et al., 2012). RLS gauges the lifespan of proliferating cells (e.g., stem cells) by tracking the production of daughter cells from a single virgin mother cell. Conversely, CLS assesses postmitotic cell aging in multicellular organisms by measuring cell survival duration in a nondividing state.

Yeast model‐based aging studies have revealed diverse conserved lifespan regulation pathways and identified an intriguing relationship between aging and nuclear mRNA export (Park et al, & Ryu, 2022). Post‐transcriptional nuclear events are closely coupled with the transcription pathway because of intricate interactions between essential factors such as TREX, TREX‐2, and nuclear pore complexes with various transcription factors and premRNA processing factors (Garcia‐Oliver et al., 2012; Rodriguez‐Navarro, 2009). Disruption of factors involved in nuclear mRNA export causes anomalous nuclear mRNA accumulation, thereby impairing the normal lifespan and vegetative growth and contributing to neurodegenerative disease pathogenesis (Lim et al., 2021, 2022; Park et al., 2022). Moreover, cellular aging disrupts the optimal conditions for efficient mRNA export, ultimately compromising the nuclear permeability barrier (Cho & Hetzer, 2020; Janssens et al., 2015; Rempel et al., 2019).

The mRNA surveillance process ensures correct processing of transcripts within the cell, coupling them to the mRNA export pathway (Choi et al., 2021; Hieronymus et al., 2004). Defects in this process can lead to cellular senescence (Son & Lee, 2017). For example, reduced RNA turnover due to decreased RNA exosome activity or oxidative stress can induce cellular senescence (Mullani et al., 2021). In chronologically aged yeast cells, the expression of *PHO84* is repressed by the corresponding antisense RNA, which is stabilized by the Rrp6 exosome complex responsible for degrading aberrant and nonfunctional RNA molecules (Camblong et al., 2007). Transcription factor Nrd1, which is involved in terminating transcription and processing specific RNAs, facilitates Rrp6 complex recruitment, thereby contributing to the maintaining the quality and quantity of RNA transcripts within the cell (Fox & Mosley, 2016). A direct correlation between Nrd1 and aging is not yet established, but the intricate interplay between Nrd1, Rrp6, and the RNA surveillance pathway may be closely linked to the cellular aging process.

The THO complex is a conserved multisubunit assembly comprising Hpr1, Tho2, Mft1, Thp2, and Tex1 that is intricately connected to other factors such as TREX and TREX‐2, which are essential for mRNA export (Vinciguerra & Stutz, 2004). Functionally, the THO complex serves as a crucial link between the transcriptional and post‐transcriptional stages of gene expression, ensuring the precise orchestration of mRNA metabolism (Strambio‐De‐Castillia et al., 2010). In *Drosophila melanogaster*., THO complex mutations reduce lifespan and stress sensitivity and are alleviated by enhanced c‐Jun N‐terminal kinase signaling for stress tolerance and longevity (Kim et al., 2011). Loss of Hpr1, a THO component, reduces RLS that is accompanied by increased genomic instability but does not increase formation of extrachromosomal rDNA circles (ERCs), a common feature of aged yeast cells (Merker & Klein, 2002). Since *hpr1Δ* cells lack ERC accumulation, which is typically linked to increased genomic instability, Hpr1 may regulate lifespan via a mechanism distinct from previously described lifespan control pathways. The other yeast subunits of the THO complex have not been analyzed from an aging‐related perspective, and knowledge is therefore limited about their direct correlation with aging.

Herein, we unveil that the loss of Hpr1 or Tho2 shortens RLS in yeast cells, independently of the well‐established RLS regulator Sir2. Rrp6 deficiency effectively rescues RLS defects triggered by artificially induced aberrant transcripts but fails to mitigate the lifespan impairment arising from Hpr1 loss. Similarly, the *nrd1‐51* and *nrd1‐102* mutations both alleviate the compromised RLS caused by the manipulation of aberrant transcripts. Notably, the presence of Nrd1 mutants, which contain an intact domain responsible for RNA polymerase II interaction, significantly ameliorate the shortened lifespan in *tho2Δ* cells. Furthermore, the interaction between Nrd1 and its target loci is augmented by the absence of Tho2, and Nrd1 overexpression exerts a negative effect on RLS, suggesting that RLS reduction mediated by Tho2 deficiency is attributed to the heightened chromatin binding of Nrd1. This phenomenon is counterbalanced by competition between normal and aberrant forms of Nrd1. Genome‐wide ChIP‐seq analysis revealed that Nrd1 is primarily recruited to genes associated with translation, and such Nrd1 recruitment is considerably enhanced by the loss of Tho2. In particular, genes associated with aging that encompass translation and RNA metabolism emerged as targets of both Tho2 and Nrd1, implying that genes that exhibit changed expression in *tho2Δ* mutants could be subject to Nrd1‐dependent transcriptional regulation. Collectively, our findings propose a model whereby the Tho2‐mediated guidance of Nrd1 modulates cellular lifespan through transcriptional control of aging‐related genes.

## 2 EXPERIMENTAL PROCEDURES

### 2.1 Yeast strains and plasmids

Yeast strains used in this study are listed in Table S1. Yeast transformation was performed using the standard lithium acetate method. To generate individual TAP‐tagged and deletion strains, the C‐terminal insertion cassette for TAP‐tagging target genes and the disruption cassette for gene deletion were constructed via PCR amplification using genomic DNA from the corresponding strain from Open Biosystems or Euroscarf. The *HIS3MX6* module derived from pFA6a‐*HIS3MX6* (Longtine et al., 1998) was employed to generate other gene deletion strains. Transformants were selected on plates of yeast extract peptone dextrose (YPD) containing G418 (200 mg/L) or synthetic complete (SC) media lacking histidine. All deletion strains were verified by PCR. The plasmids used in this study were previously described (Honorine et al., 2011; Longtine et al., 1998), and are listed in Table S2.

### 2.2 Spotting assay

The spotting assay was performed as described previously (Lim et al., 2022; Ryu & Ahn, 2014). Exponentially growing liquid cultures were normalized to 0.1 OD_600_ and diluted in a 10‐fold series. Then, 5 μL of each dilution was spotted onto YPD plates, unless otherwise indicated, and the plates were incubated at 25, 30, or 37°C for 2–5 days. For doxycycline treatment, 5 μL of each dilution were spotted onto the SC plate with or without doxycycline (1 μg/mL), and the plates were incubated at 25°C for 2–3 days.

### 2.3 RLS analysis

Yeast strain RLS was measured on YPD plates unless otherwise indicated, as described previously (Lim et al., 2022; Ryu et al., 2014). The lifespan of approximately 50 virgin daughter cells was analyzed. To assess lifespan differences, a Mann–Whitney test was performed with a cutoff of *p* = 0.05; the average lifespan was considered different at *p* < 0.05 (Kaeberlein et al., 1999). The comparison values between the experimental‐matched WT and each mutant are listed in Table S3.

### 2.4 CLS analysis

Yeast strain CLS was measured as previously described (Lim et al., 2021). Briefly, an overnight culture was diluted to 0.01 OD_600_ into fresh SC medium to a final volume of 10 mL (with a flask‐to‐culture volume of 5:1) and incubated at 30°C with shaking (200 rpm). Cells were spread on duplicate YPD plates at the indicated time points with 10 μL of 1:1000 diluted culture and 100 μL of autoclaved distilled water. YPD plates were incubated at 30°C, and viability was assessed after 2–3 days with the average colony forming unit (CFU) count. The spreading step was repeated every 1 or 2 days until the end of the experiment. GeneSnap and GeneTools software (Syngene) were used to image plates and calculate CFUs, respectively. The days required for populations to reach 50% and 10% viability were calculated as representations of median (50%) and maximum (10%) lifespans to facilitate comparisons between WT and mutant strains.

### 2.5 ChIP‐seq and data analysis

ChIP assay of Nrd1‐TAP‐tagged strains was performed as described previously (H. Y. Ryu et al., 2016; 2019; 2020). Briefly, formaldehyde was added to mid‐log phase cells to a final concentration of 1% for 20 min. Crosslinking was quenched by addition of glycine to 240 mM. Crosslinked cells were collected via centrifugation, washed twice in TBS, and then lysed with glass beads in FA lysis buffer (50 mM HEPES–KOH [pH 7.5], 150 mM NaCl, 1 mM EDTA, 1% Triton X‐100, 0.1% Na deoxycholate, 0.1% SDS, protease inhibitor cocktail [Roche, 11697498001], and 1 mM PMSF [AmericanBio, AB01620]). Sonicated chromatin was incubated with IgG‐Sepharose beads (GE Healthcare, 17–0969‐01). Precipitates were sequentially washed with FA lysis buffer with 275 mM NaCl, FA lysis buffer with 500 mM NaCl, LiCl washing buffer (10 mM Tris–HCl [pH 8.0], 0.1 mM EDTA, 250 mM LiCl, 0.5% NP‐40, and 0.5% sodium deoxycholate) and TE (10 mM Tris–HCl [pH 8.0], and 1 mM EDTA), and then eluted with elution buffer (10 mM Tris–HCl [pH 7.5], 10 mM EDTA, and 1% SDS) at 65°C. Eluted chromatin fragments were treated with pronase (Roche, 11459643001), and then DNA was purified via phenol/chloroform extraction. Diluted template DNA (1:10 and 1:1000 dilution for IP and input DNA, respectively) was used in qPCR reactions using AccuPower® 2X GreenStar™ qPCR Master Mix (Bioneer, K6254) on the CFX Connect Real‐Time PCR System (Bio‐Rad); signals were normalized to the internal control (a fragment amplified from an untranscribed region on ChrIV, residues 1516109–1516234; SGD) and input DNA.

Sequencing libraries were constructed utilizing the TruSeq DNA Sample prep kit according to manufacturer's protocols (TruSeq ChIP Sample preparation guide 15023092 Rev. B) and sequenced using 100 bp paired‐end sequencing on an Illumina NovaSeq 6000 following manufacturer's protocols. Reads were trimmed using Trimmomatic (v0.38) and aligned to the *S*. *cerevisiae* reference genome (sacCer3) using BowTie (v1.1.2). Peaks were called utilizing MACS2 (v2.1.1.20160309) and duplicate reads were processed using Picard (v0.118). Called peaks were annotated using ChIPSeeker (v1.16.1) with gene models from SGD. Comparative analysis of data was performed using csaw (v1.34.0). The normalized bedGraph files were generated using MACS2 and then converted to bigwig files using the bedGraphToBigWig program. The genome‐wide profile was generated using the ‘computeMatrix scale‐regions’, ‘plotProfile,’ and ‘plotHeatmap’ tools in the deepTools package (v3.1.3). All GO analyses were performed utilizing the Database for Annotation, Visualization and Integrated Discovery (DAVID) database. All GO was filtered by the EASE score, a modified Fisher's Exact *p*‐value utilized by the DAVID database, with an EASE score less than 0.1. GO plots were created with the ggplot2 (v3.4.4) R library. ChIP‐seq tracks were viewed in the Integrative Genomics Viewer (IGV, https://igv.org/).

### 2.6 RNA‐seq and data analysis

Yeast strains WT (BY4741) and *tho2Δ* (FY254) were grown in 5 mL of YPD until mid‐log phase. The hot phenol method was used to prepare RNA from a 1.0 OD_600_ equivalent of cells as previously described (Ryu et al., 2014; 2018). Construction of libraries and subsequent sequencing was performed by Macrogen (Seoul, Korea) with duplicate RNA samples. GO enrichment analyses were performed using Panther 17.0 (http://www.pantherdb.org/). Volcano plots were drawn using R studio with the ggplot2 package.

### 2.7 Re‐analysis of Nrd1 PAR‐CLIP data

To re‐analyze Nrd1‐bound genes, previous data from photoactivatable ribonucleoside‐enhanced crosslinking and immunoprecipitation (PAR‐CLIP) was downloaded from the Gene Expression Omnibus database (GSM791764) (Jamonnak et al., 2011). Adapter sequences were removed from fastq files with cutadapt (Martin, 2011). All reads were mapped to *S*.*cerevisiae* genome (Ensembl version 107) with bowtie2 (Langmead et al., 2019). Binding sites were identified and annotated with PEAKachu (https://github.com/tbischler/PEAKachu).

## 3 RESULTS

### 3.1 THO complex regulates RLS in a Sir2‐independent manner

**FIGURE 1:**
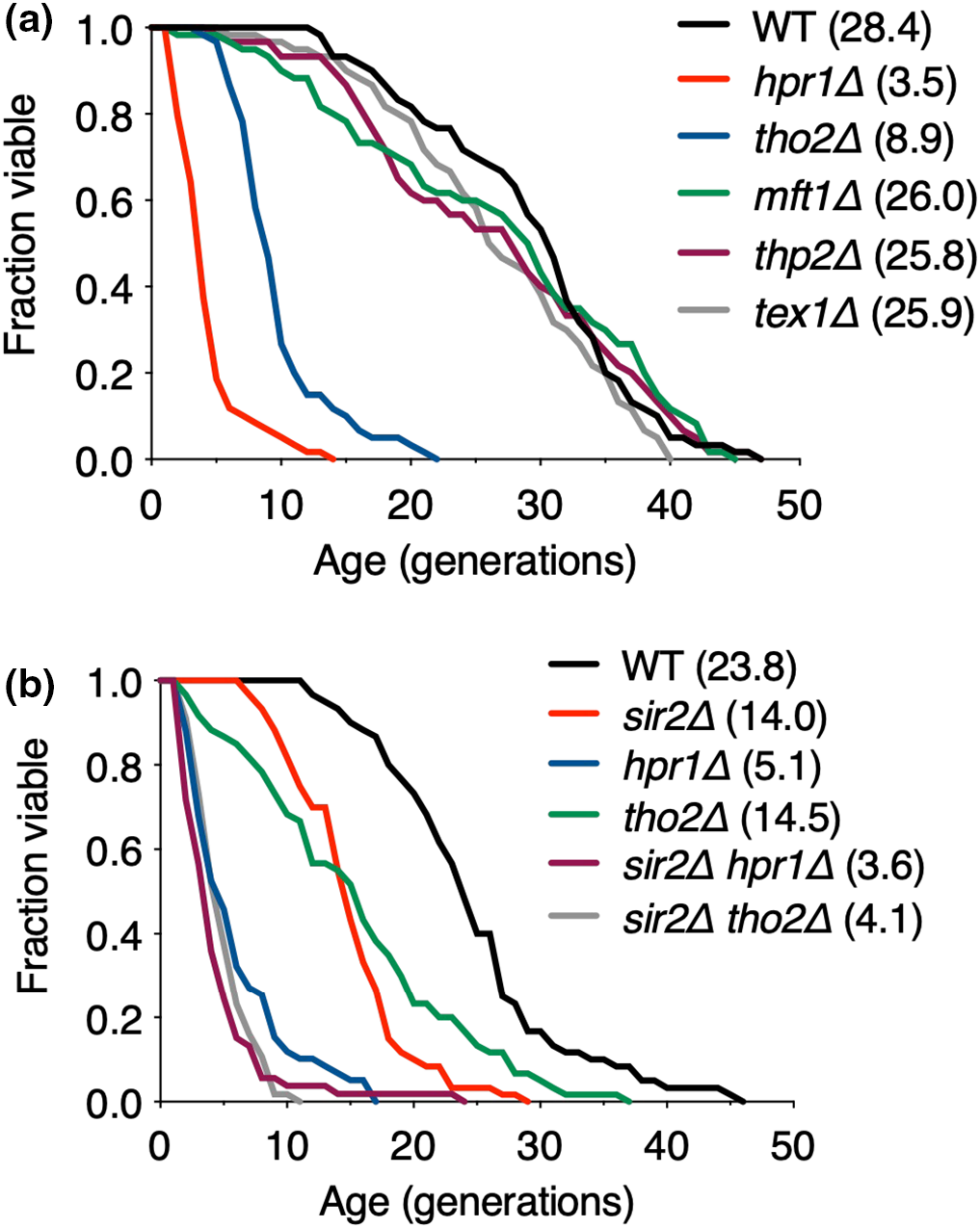
Hpr1 and Tho2 in THO complex affect RLS in a Sir2‐independent manner. (a, b) RLS analysis of WT and the indicated mutants. The mean lifespans are shown in parentheses.

We recently reported that defects in the TREX‐2 complex, which is physically and functionally linked to the THO complex, impair RLS via aberrant mRNA nuclear export (Lim et al., 2022). Therefore, to investigate whether the THO complex is involved in the control of yeast lifespan, we first analyzed the RLS of yeast cells that lacked individual subunits of THO complex: Hpr1, Tho2, Mft1, Thp2, or Tex1 (Figure 1a). Those cells lacking Hpr1 and Tho2, the two most prominent components of THO complex, exhibited markedly reduced RLS compared with that of the wild‐type (WT) cells, whereas the absence Mtf1, Thp2, and Tex1, did not impact RLS, suggesting that Hpr1 and Tho2, which are the only subunits conserved from yeast to humans (Park et al., 2022), appear to be essential for regulating lifespan.

We next addressed how the THO complex is involved in lifespan regulation. As Sir2 is a recognized modulator of RLS (Dang et al., 2009; Kaeberlein et al., 1999; Sinclair & Guarente, 1997), we subsequently investigated whether Sir2 affects RLS in *hpr1Δ* or *tho2Δ* mutants (Figure 1b). The absence of Hpr1 or Tho2 substantially exacerbated the shortened lifespan of *sir2Δ* cells, suggesting that the THO complex contributes to lifespan modulation through a Sir2‐independent pathway.

### 3.2 Hpr1 affects lifespan independently of exonuclease component Rrp6

**FIGURE 2:**
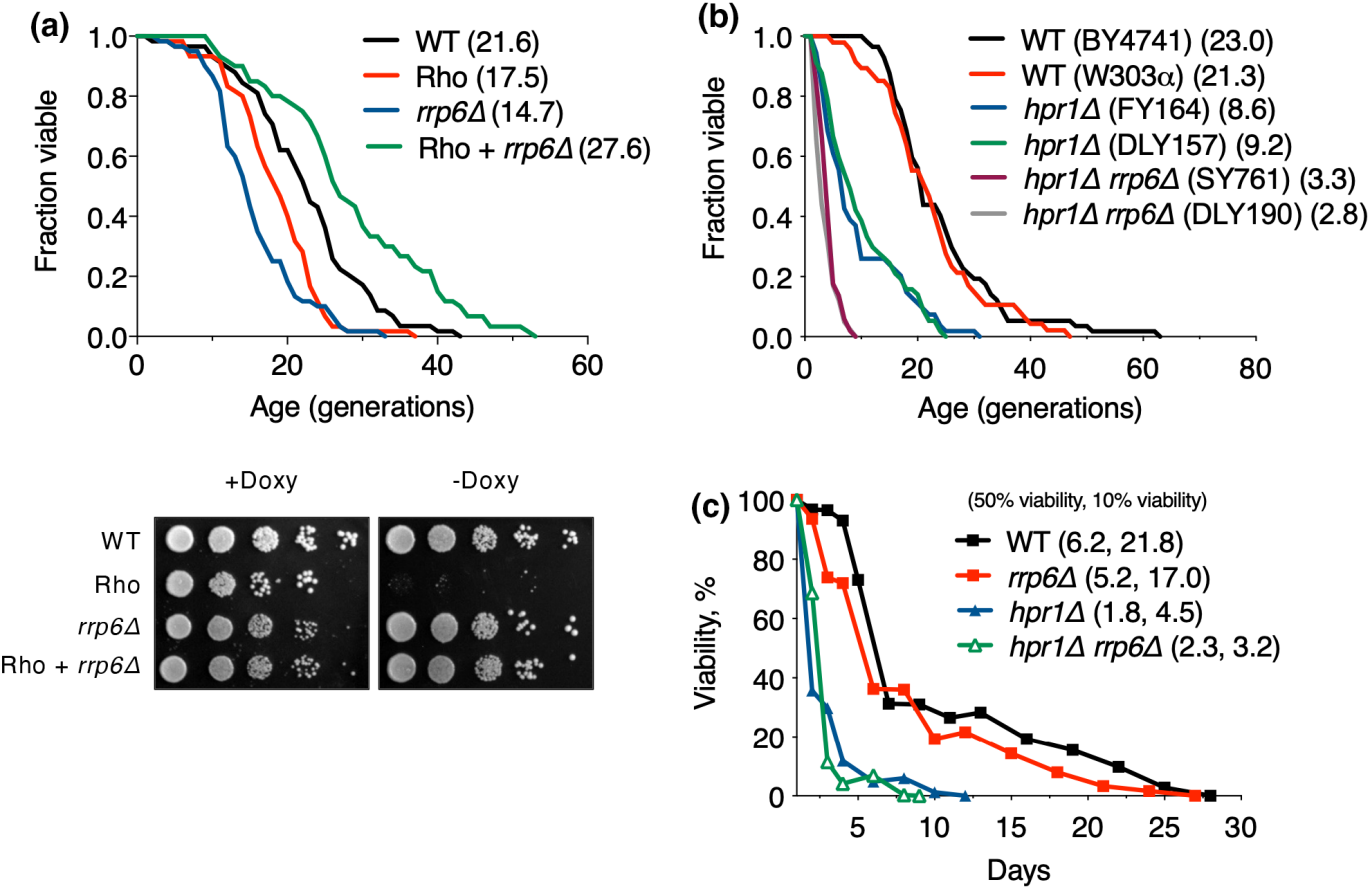
*RRP6* deletion rescues impaired RLS in Rho‐induced cells, but not *hpr1Δ* cells. (a) RLS analysis of WT and *rrp6∆* strains transformed with empty vector (pCM189) or plasmid‐expressing Rho (pCM189‐Rho), as described in Figure 1. For the growth analysis, tenfold dilutions of the indicated strains were spotted onto Rho‐repressing (+Doxy) or Rho‐inducing (−Doxy) plates and then grown at 25°C for 2–3 days. (b) RLS analysis of WT and the indicated mutant strains in two different genetic backgrounds (BY4741 and W303α), as described in Figure 1. (c) CLS analysis of WT and the indicated mutants. The two numbers in parentheses indicate the median and maximum lifespans, calculated as the day when cultures reached 50% and 10% viability, respectively.

The THO complex has a pivotal role in mRNP biogenesis by bridging transcription and mRNA maturation and export (Strambio‐De‐Castillia et al., 2010). Deletion of *HPR1* or *MFT1* causes accumulation of nuclear poly(A)^+^ RNA, whereas the loss of Rrp6, a nuclear exosome component, restores a semi‐normal level of full‐length transcripts and reverses the transcript sequestration phenotype in *hpr1Δ* or *tho2Δ* mutants (Libri et al., 2002). In addition, the expression of Rho, a bacterial transcription termination factor, in yeast disrupts normal mRNP biogenesis with aberrant transcripts produced that are degraded by the Rrp6 complex together with the occurrence of significant growth defects (Honorine et al., 2011). Thus, to determine whether abnormal accumulation of nuclear RNA affects yeast lifespan, we performed RLS analysis in cells expressing Rho (Figure 2a). As anticipated, Rho‐expressing cells exhibited a decrease in RLS and growth, which was totally alleviated by Rrp6 deficiency, suggesting that the retention of defective transcripts negatively affects cellular lifespan. However, although RLS was also impaired in other exosome‐related factor mutants, *trf4Δ*, *xrn1Δ*, or *sen1‐1*, mutation of *trf4* or *sen1* did not rescue the Rho‐induced RLS defects (Figure S1), implying the Rrp6 complex may be a primary RNA‐degradation controller of lifespan.

We next investigated whether Hpr1‐dependent lifespan shortening is governed by Rrp6‐driven pathway (Figure 2b). Interestingly, the altered RLS of *hpr1Δ rrp6Δ* double mutant significantly exceeded that of *hpr1Δ* single mutant, suggesting that Hpr1 influences yeast lifespan through a mechanism independent of Rrp6. Further insight into the role of Hpr1 in lifespan control was gained through CLS analysis in *hpr1Δ*, *rrp6Δ*, and *hpr1Δ rrp6Δ* strains (Figure 2c). In agreement with the RLS analysis, the life span of *hpr1Δ* or *rrp6Δ* strains was significantly reduced to a maximum of 4.5 or 17.0 days, respectively, compared with 21.8 days of WT cells, and *hpr1Δ rrp6Δ* strain displayed a more severe lifespan defect than that in null *rrp6* mutant. Taken together, these results suggest that Rrp6‐mediated RNA surveillance did not circumvent the Hpr1 loss‐induced RLS and CLS defects.

### 3.3 Nrd1 mutations restore rho‐induced shortened RLS

**FIGURE 3:**
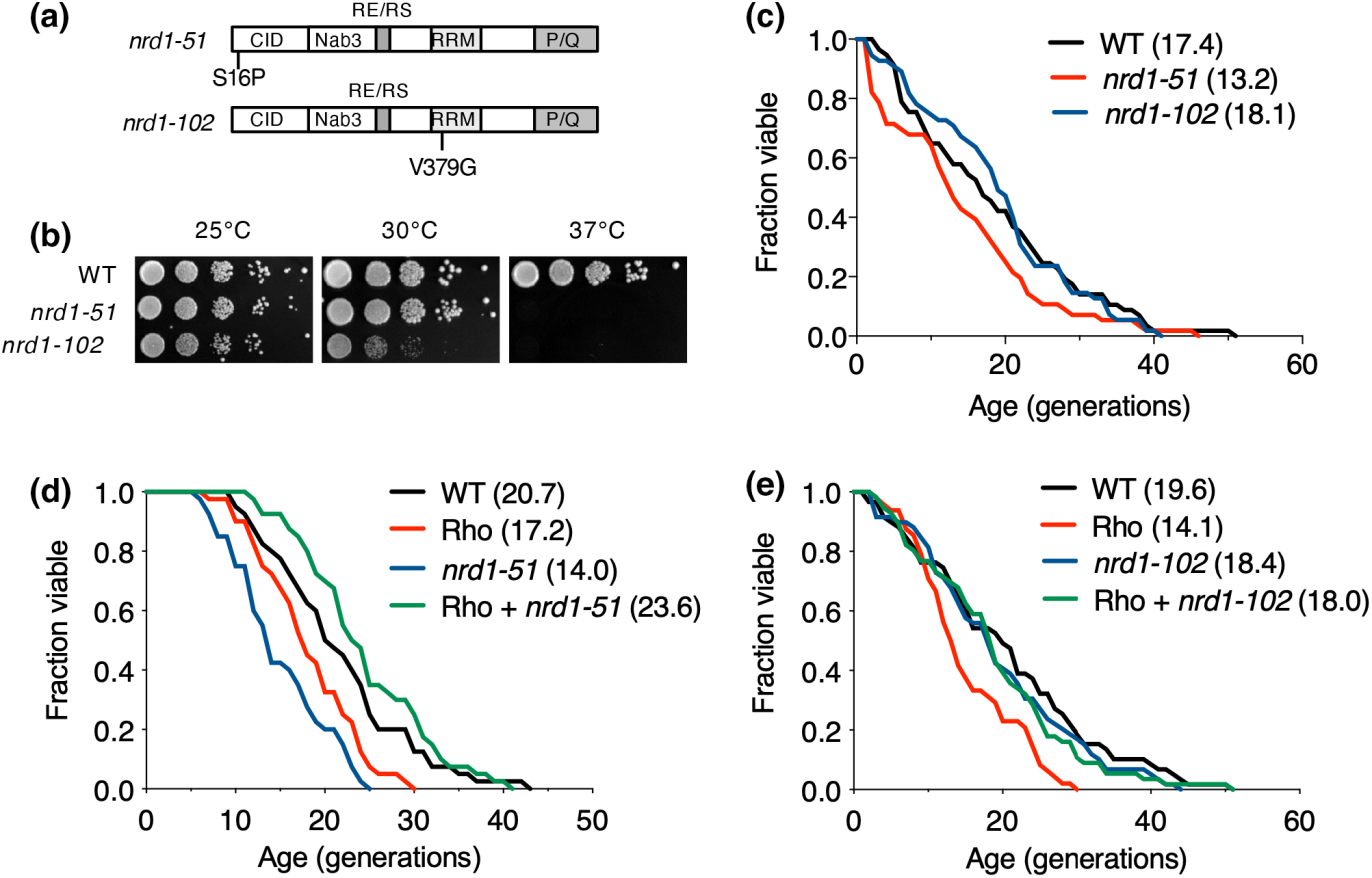
Expression of Rho in Nrd1 mutants restore shortened RLS. (a) Schematic diagrams of *nrd1* temperature‐sensitive alleles, *nrd1‐51* (S16P) and *nrd1‐102* (V379G). Nrd1 domains include RNA polymerase II CTD‐interacting domain (CID), Nab3 interaction domain (Nab3), arginine‐glutamine and arginine‐serine‐rich region (RE/RS), RNA recognition motif (RRM), and proline‐ and glutamine‐rich region (P/Q). (b) Growth analysis of *nrd1‐51* and *nrd1‐102* strains on YPD plates at 25, 30, and 37°C, as described in Figure 2a. (c–e) RLS analysis of WT and indicated mutants transformed without (c) or with pCM189 or pCM189‐Rho (d, e), as described in Figure 1.

Rho expression promotes Rrp6 and Nrd1cotranscriptional recruitment, which is necessary for the 3′‐end processing of specific RNAs (Honorine et al., 2011). Accordingly, we tested whether the Rho‐induced abnormal lifespan is connected to the role of Nrd1. Consistent with previous results in *nrd1* temperature‐sensitive mutants (Conrad et al., 2000), we observed strong sensitivity to 37°C in both *nrd1‐51* and *nrd1‐102* strains bearing mutations in the CTD‐interaction domain (CID) and RNA recognition motif (RRM) of Nrd1, respectively (Figure 3a,b). Moreover, RLS was significantly decreased in *nrd1‐51*, but not in *nrd1‐102* (Figure 3c). Similar to the effect of additional deletion of *RRP6* on RLS in Rho‐expressing cells, either of these mutations rescued the impaired RLS induced by Rho activation (Figure 3d,e), suggesting that Nrd1, which interacts with Rrp6, contributes to cellular lifespan possibly by controlling the 3′ end formation for degradation of target transcripts.

### 3.4 Nrd1 truncated variants alleviate the lifespan defect in *tho2Δ* cells

**FIGURE 4:**
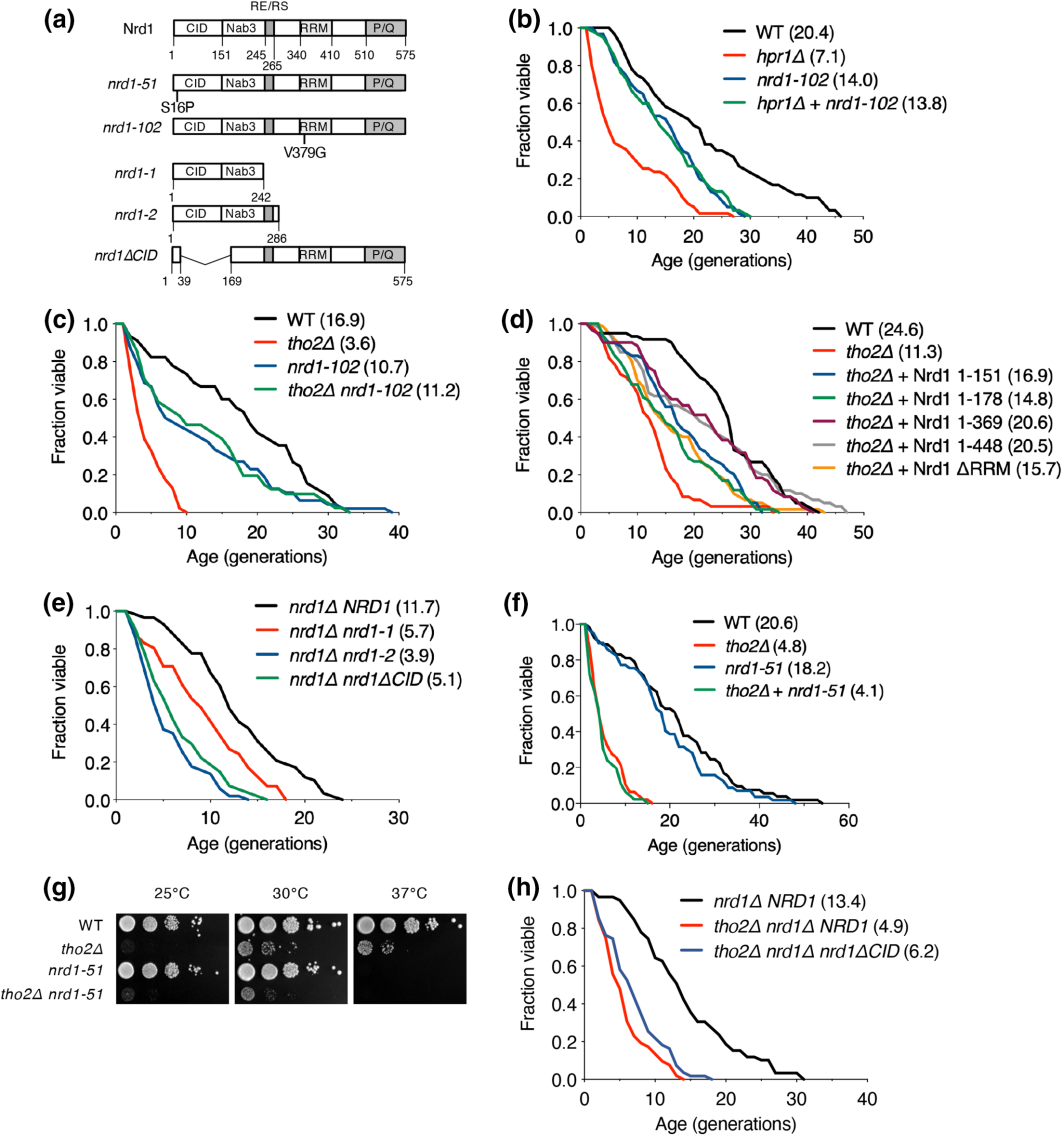
Overexpression of Nrd1 truncates significantly rescues impaired RLS in *tho2Δ* cells. (a) Schematic diagrams of Nrd1 point mutants and truncates. (b, c, f) RLS analysis of WT and the indicated mutants, as described in Figure 1. (d) RLS analysis of WT or *tho2Δ* strains transformed with empty vector (pAG425GPD) or plasmids expressing indicated Nrd1 truncated variants, as described in Figure 1. (e, h) RLS analysis of *nrd1Δ* or *tho2Δ nrd1Δ* strains transformed with a plasmid‐expressing *NRD1* or *nrd1ΔCID*, as described in Figure 1. (g) Growth analysis of strains used in (f) on YPD plates at 25, 30, and 37°C, as described in Figure 2a.

Nrd1 links the transcription termination process with the surveillance pathway of the exosome. However, Rrp6 is involved in Nrd1‐independent processing and degradation of noncoding RNA (ncRNA) (Fasken et al., 2015), and their interplay remains incompletely understood. Although our results suggest that Hpr1 affects both RLS and CLS through a pathway independent of Rrp6 (Figure 2b,c), Nrd1 may directly participate in regulating lifespan via the THO complex. Therefore, we investigated whether Hpr1 and Tho2, components of the THO complex‐dependent lifespan shortening is determined by a Nrd1‐driven pathway (Figure 4b,c). Interestingly, the additional *nrd1‐102* mutation significantly rescued the shortened lifespan in cells lacking Hpr1 or Tho2, suggesting that Nrd1 and Rrp6 are differentially involved in the context of lifespan control by the THO complex.

Next, to understand which domain of Nrd1 is crucial for relieving RLS defects in *tho2Δ* cells, we assessed the RLS change in *tho2Δ* strains transformed with vectors that expressed truncated Nrd1 proteins of residues 1–151, 1–178, 1–369, or 1–448, or lacking RRM (Figure 4d). The additional expression of most Nrd1 truncates substantially restored the reduced RLS of the *tho2Δ* strain. Since the endogenous *NRD1* gene was still expressed in *tho2Δ* strain expressing the Nrd1 truncates, the disturbance of WT Nrd1 function by the presence of Nrd1 truncates may be a key element for recovery of RLS impairment caused by Tho2 loss. Furthermore, introducing the Nrd1 truncates resulted in a significant improvement in the diminished RLS observed in the *hpr1Δ* strain (Figure S2). In addition, RLS was considerably decreased in the *nrd1ΔCID* mutant (Figure 4e), and neither *nrd1*–*51* nor *nrd1ΔCID* mutations improved the shortened lifespan or growth defects in the *tho2Δ* strain (Figure 4f–h), implying that binding of Nrd1 to RNA polymerase II CTD is important for lifespan regulation. Similarly, mutation of the cleavage polyadenylation factor Fip1 or poly(A) polymerase Pap1, involved in the general mRNA termination factors (Zhang et al., 2012), failed to restore RLS severity in the *tho2Δ* strain, suggesting that general 3′‐end processing factors, Fip1 and Pap1, affect lifespan in a Tho2‐dependent manner (Figure S3). Overall, these results strongly suggested that the interaction of Nrd1 with RNA polymerase II is necessary for the THO complex‐dependent lifespan control pathway.

### 3.5 Nrd1 regulates lifespan through Tho2‐dependent pathway

**FIGURE 5:**
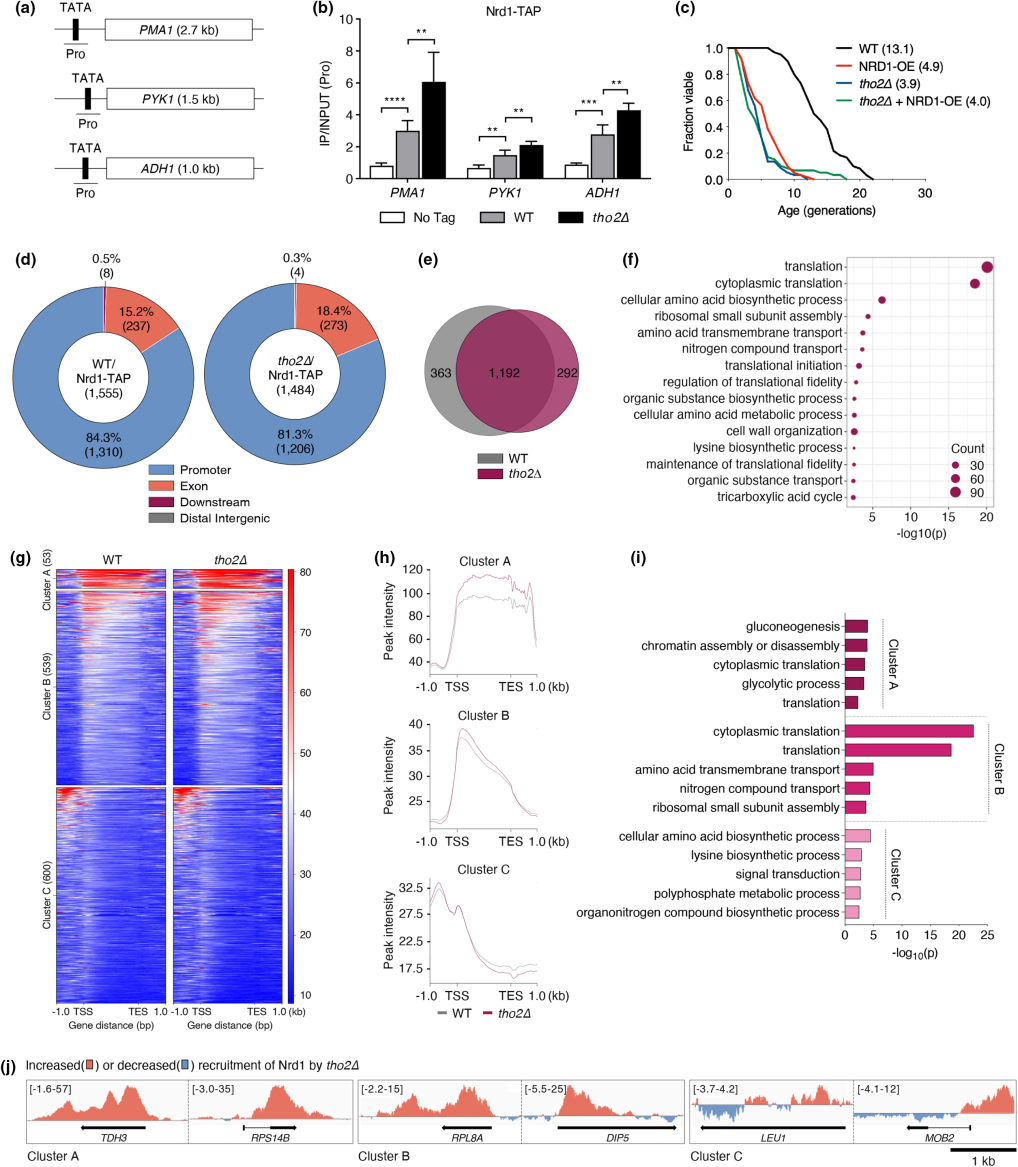
Overexpression of Nrd1 impairs RLS in a Tho2‐dependent manner. (a) Schematic diagram of *PMA1*, *PYK1*, and *ADH1* genes. The TATA/promoter (Pro) and ORF are represented by black and white boxes, respectively. Bars below the genes show the relative positions of the PCR products used in the ChIP‐qPCR analysis. (b) ChIP analysis using IgG‐sepharose beads in indicated strains expressing TAP‐tagged Nrd1. The BY4741 (no tag) strain was used as a negative control. qPCR signals of the indicated genes were quantitated and normalized to an internal background control and the input DNA. Primer pairs used are indicated in (a). Error bars indicate SDs calculated from four independent chromatin preparations. Asterisks indicate statistically significant differences between the corresponding samples using a two‐tailed Student's *t* test (*p < 0.05; **p < 0.01; ***p < 0.001; ****p < 0.0001). (c) RLS analysis of WT and *tho2Δ* strains transformed with pAG425GPD or pAG425GPD‐NRD1 (NRD1‐OE), as described in Figure 1. (d) Summary of ChIP‐seq data of Nrd1‐TAP in WT and *tho2Δ* strains. The pie charts show the distribution of Nrd1 peaks at promoter, exon, downstream, and distal intergenic regions in WT (left panel) and *tho2Δ* (right panel) strains. Numbers in parentheses indicate the number of detected sites. (e) Venn diagram of Nrd1 peaks displayed in (d). (f) Dot plot of GO enrichment analysis for the Nrd1‐enriched genes in WT. Dot size represents the number of genes in the significant gene list associated with the GO term. The *x*‐axis corresponds to the negative logarithm of the *p*‐value. (g) Clustered heatmap of ChIP‐seq data from (d). Nrd1‐bound peaks across the gene with substantial enrichment at transcription start site (TSS) and transcript end sites (TES) are separated into three clusters by *K*‐means clustering. Numbers in parentheses indicate the number of detected Nrd1 peaks. (h) ChIP read‐density profile of (g). The profile plots show the average ChIP signals of each cluster. (i) GO enrichment analysis of the genes in (g). Bar diagrams indicate the fold‐enrichment of GO biological process of each cluster. (j) Integrative Genomics Viewer (IGV) screenshot of ChIP‐seq data showing increased (red) or decreased (blue) levels of Nrd1 recruitment by *tho2Δ*. The normalized ratio of IP/INPUT peaks in WT is subtracted from the peak ratio in *tho2Δ* to illustrate the observed changes at indicated genes.

Our comprehensive assessment of RLS strongly indicated a pronounced correlation between Nrd1 and the THO complex‐mediated lifespan regulatory pathway (Figure 4). In contrast to other termination factors, such as RNA14, Nrd1 preferentially binds to the CTD serine 5 phosphorylation form of RNA polymerase II, which is crucial for transcription initiation and early stages of RNA synthesis during transcription and whose binding loci include the promoter regions of *PMA1*, *PYK1*, and *ADH1* (Heo et al., 2013; Vasiljeva et al., 2008). In addition, the THO complex, including Tho2, is recruited to constitutively expressed genes, such as *PMA1*, and subsequently cotraverses the open reading frame (ORF) in tandem with RNA polymerase II (Luna et al., 2005; Strasser et al., 2002). Therefore, to more fully understand the intricate interplay between Nrd1 and the THO complex, we used chromatin immunoprecipitation (ChIP) to monitor Nrd1 recruitment at *PMA1*, *PYK1*, and *ADH1* promoter regions in *tho2Δ* cells (Figure 5a,b). Remarkably, Nrd1 association with constitutive genes was significantly increased in *tho2Δ* cells, suggesting that Tho2 serves as a general regulator, impeding aberrant Nrd1 recruitment to chromatin during transcription.

These ChIP results provided supporting evidence for our hypothesis that the aberrant Nrd1 functioning competes with the endogenous normal Nrd1 in *tho2Δ* cells, resulting in RLS recovery (Figure 4b). As the elevated recruitment of Nrd1 due to Tho2 loss was detrimental to RLS but was counterbalanced by competition with the introduction of aberrant forms of Nrd1, we next conducted RLS analysis in cells overexpressing Nrd1 to validate our hypothesis (Figure 5c). Nrd1 overexpression indeed impaired normal RLS, and this defect remained unaffected by additional deletion of *THO2*, suggesting that the effects of Nrd1 on RLS rely on the presence of Tho2. Taken together, these results strongly suggest that the THO complex is necessary for a cellular lifespan by preventing aberrant recruitment of Nrd1 to chromatin.

### 3.6 Nrd1 recruitment is notably increased at the translation‐associated genes in *tho2Δ* cells

To map the sites of augmented Nrd1 recruitment resulting from Tho2 deficiency on a genome‐wide scale, we carried out ChIP‐seq experiments (Figure 5d,e). Nrd1 exhibited predominant enrichment in various promoter (more than 80%) and exon (more than 15%) regions in both WT and *tho2Δ* strains (see Figure 5d), which is in agreement with the previously reported results (Gudipati et al., 2008; Vasiljeva et al., 2008). Notably, Gene Ontology (GO) analysis revealed that WT Nrd1 is primarily recruited to genes associated with translation (Figure 5f). Next, to classify gene sets under Tho2‐mediated regulation of Nrd1 recruitment, we performed *K*‐means cluster analysis of Nrd1 ChIP‐seq results (Figure 5g,j) and identify three clusters with distinct patterns (Figure 5h): increased recruitment of Nrd1 from transcription start site (TSS) to transcript end sites (TES) in *tho2Δ* (cluster A), increased recruitment of Nrd1at TSS in *tho2Δ* (cluster B), and decreased recruitment of Nrd1 at TES in *tho2Δ* (cluster C). Interestingly, GO analysis of cluster genes indicated that genes related to translation are include in gene groups of clusters A and B, suggesting that translation‐associated genes are major targets of Tho2‐mediated surveillance to curb the excessive recruitment of Nrd1.

### 3.7 Genes related to translation and nuclear transport are downregulated in both *tho2Δ* and aged cells

**FIGURE 6:**
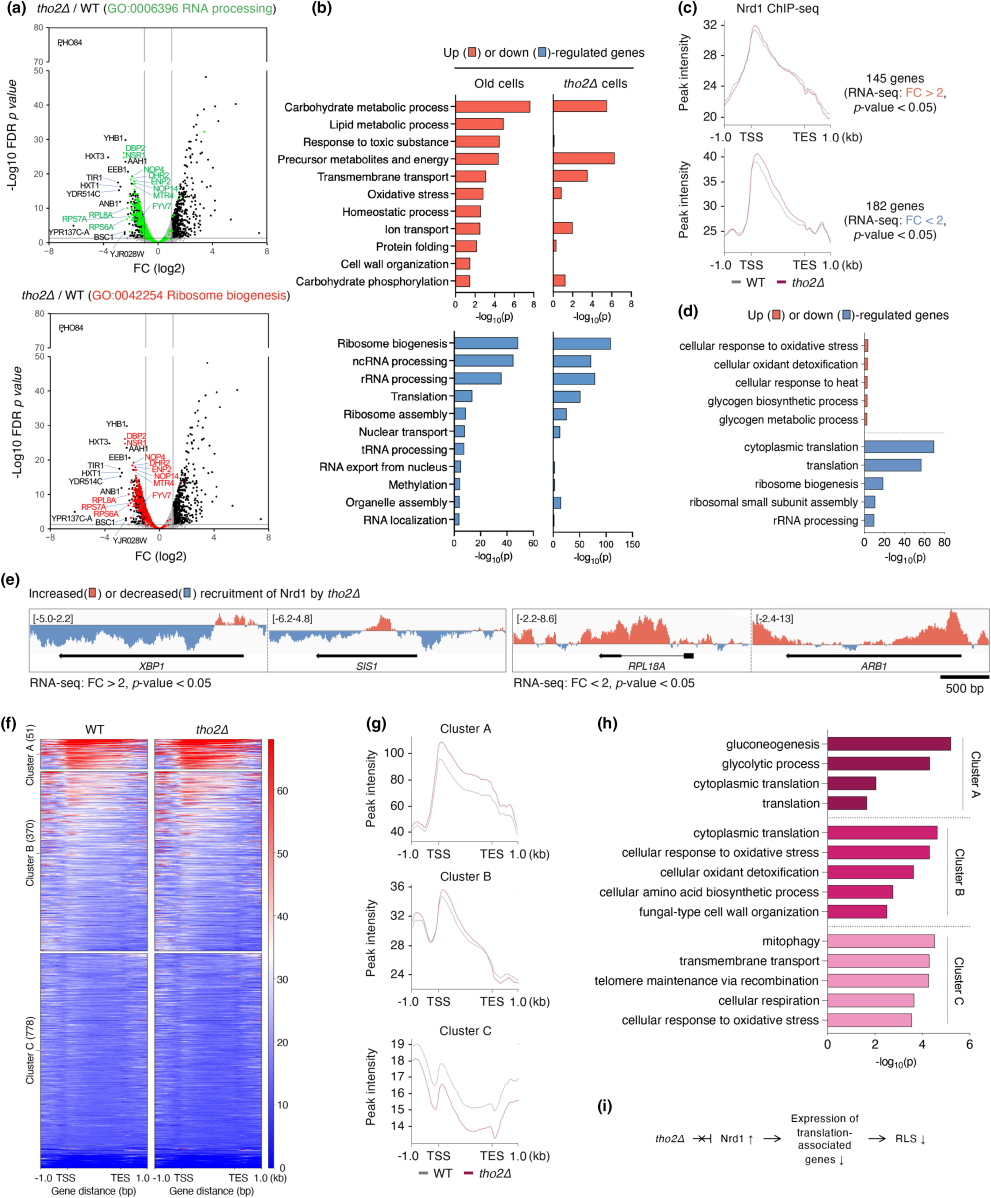
Comparison of mRNA profiles between *tho2Δ* and aged cells. (a) Volcano plots displaying differentially expressed genes in the *tho2Δ* strain as compared with the WT. The *y*‐axis is the mean of the negative logarithm of the *p*‐values, and the *x*‐axis corresponds the log2 fold change value. Gray horizontal and vertical lines indicate a *p*‐value of 0.05 and log2 fold change of −2 and 2, respectively. Green and red dots represent genes enriched in GO terms related to RNA processing and ribosome biogenesis, respectively. (b) GO enrichment analysis of the genes in old cells from previous RNA‐seq experiments (Hendrickson et al., 2018) and displayed in (a). Lists of differentially expressed genes in old cells compared with young cells were acquired by filtering the data by *p*‐value of ≤0.05 and |fold change| >1. Bar diagrams indicate the fold‐enrichment of top 11 GO biological process terms in the up (red)‐ or down (blue)‐regulated genes. (c) Nrd1 ChIP read‐density profile of differentially expressed genes in the *tho2Δ* strain as compared with the WT, as described in Figure 6h. For the analysis of up‐ and down‐regulated genes (upper and bottom panels, respectively), 145 and 182 genes were acquired by filtering the data with *p*‐value of ≤0.05 and |fold change| >2, respectively. (d) GO analysis of the up (red)‐ or down (blue)‐regulated genes in (c), as described in (a). (e) IGV screenshot of ChIP‐seq data showing altered recruitment levels of Nrd1 by *tho2Δ*, as described in Figure 5J. (f–h) Cluster analysis of Nrd1 binding peaks at the differentially expressed genes in old cells (Hendrickson et al., 2018) in WT and *tho2Δ* strains. Heatmap (f), density profile (g), and GO analysis (h) of the *K*‐means clustering of mapped read position for Nrd1 occupancy. (i) Schematic diagram depicting the role of Tho2 in the regulation of RLS. The deletion of *tho2* fails to prevent the excessive recruitment of Nrd1, resulting in decreased expression of translation‐associated genes, which in turn leads to a reduction in RLS. See text for further details.

Since our data indicated that aging closely correlates with the THO complex, which links RNA metabolism with transcription (Jimeno et al., 2002), we used RNA‐seq to analyze genome‐wide gene expression profiles of *tho2Δ* cells and compared these profiles with previously published RNA‐seq data from aged cells (Hendrickson et al., 2018) (Figure 6a,b). In accordance with the earlier transcriptome analysis of cells depleted of THO components in *D*.*melanogaster* (Rehwinkel et al., 2004), loss of Tho2 altered the expression of numerous genes (Figure 6a). Notably, GO analysis revealed that the expression of many genes related to RNA processing and ribosome biogenesis was significantly downregulated by deletion of *THO2* compared with their WT levels. Moreover, similar to the transcriptome changes observed in aged cells, the expression of translation‐related genes, such as those involving ribosome biogenesis, rRNA processing, ribosome assembly (Ryu, 2022), and nuclear transport, was significantly reduced in *tho2Δ* cells (Figure 6b). Furthermore, both *tho2Δ* and aged cells exhibited upregulated expression of genes associated with carbohydrate metabolism, precursor metabolites and energy, and transmembrane transport. Thus, our data showed that loss of Tho2 reduced expression of genes involved in translation and RNA processing, which is a pattern predominantly observed in aged cells, suggesting that a subset of genes associated with aging are targets of THO complex.

Subsequently, we compared the alterations in gene expression in tho2*Δ* strains with the ChIP‐seq results of Nrd1 presented in Figure 5d (Figure 6c). Among the 145 genes that were upregulated by more than 2‐fold relative to WT in *tho2Δ* cells, the recruitment of Nrd1 was not significantly affected by the loss of Tho2 (Figure 6c, upper panel). However, an apparent increase in Nrd1 occupancy from TSS to TES was observed in the 182 genes that were down‐regulated by 2‐fold in cells lacking Tho2 (Figure 6c, bottom panel). Additionally, GO analysis of the 2‐fold down‐regulated genes in *tho2Δ* revealed their involvement in translation processes, including ribosome biogenesis, ribosomal small subunit assembly, and rRNA processing (Figure 6d). Taken together, these findings suggest that the loss of Tho2 mediates an increase in the association of Nrd1 with translation‐related genes, such as *RPL18A* (ribosomal 60S subunit) or *ARB1* (ribosome biogenesis factor) (Figure 6e), leading to a negative impact on their gene expression.

Because our data showed that transcription termination factor Nrd1 is important for the THO complex‐dependent lifespan regulation pathway, we further compared ChIP‐seq data for Nrd1‐TAP, as shown in Figure 5d, with genes exhibiting significant expression changes in aged cells (Hendrickson et al., 2018) (Figure 6f). The *K*‐means cluster analysis of Nrd1 ChIP‐seq results categorized three clusters with different patterns. Nrd1 recruitment to genes listed in both clusters A and B was notably enhanced by the loss of Tho2 (Figure 6g, upper and middle panels), whereas cluster C did not exhibit such an increase pattern (Figure 6g, bottom panel). Moreover, GO analysis of cluster genes indicated that translation‐related genes constitute a substantial portion of genes in clusters A and B. Thus, our data strongly imply that translation‐related genes are obviously influenced by Tho2‐mediated regulation of Nrd1 pathway, and this transcription factor crosstalk is closely linked to aging (Figure 6i **)**.

### 3.8 Subsets of genes involved in translation and RNA processing are targets of both Tho2 and Nrd1

**FIGURE 7:**
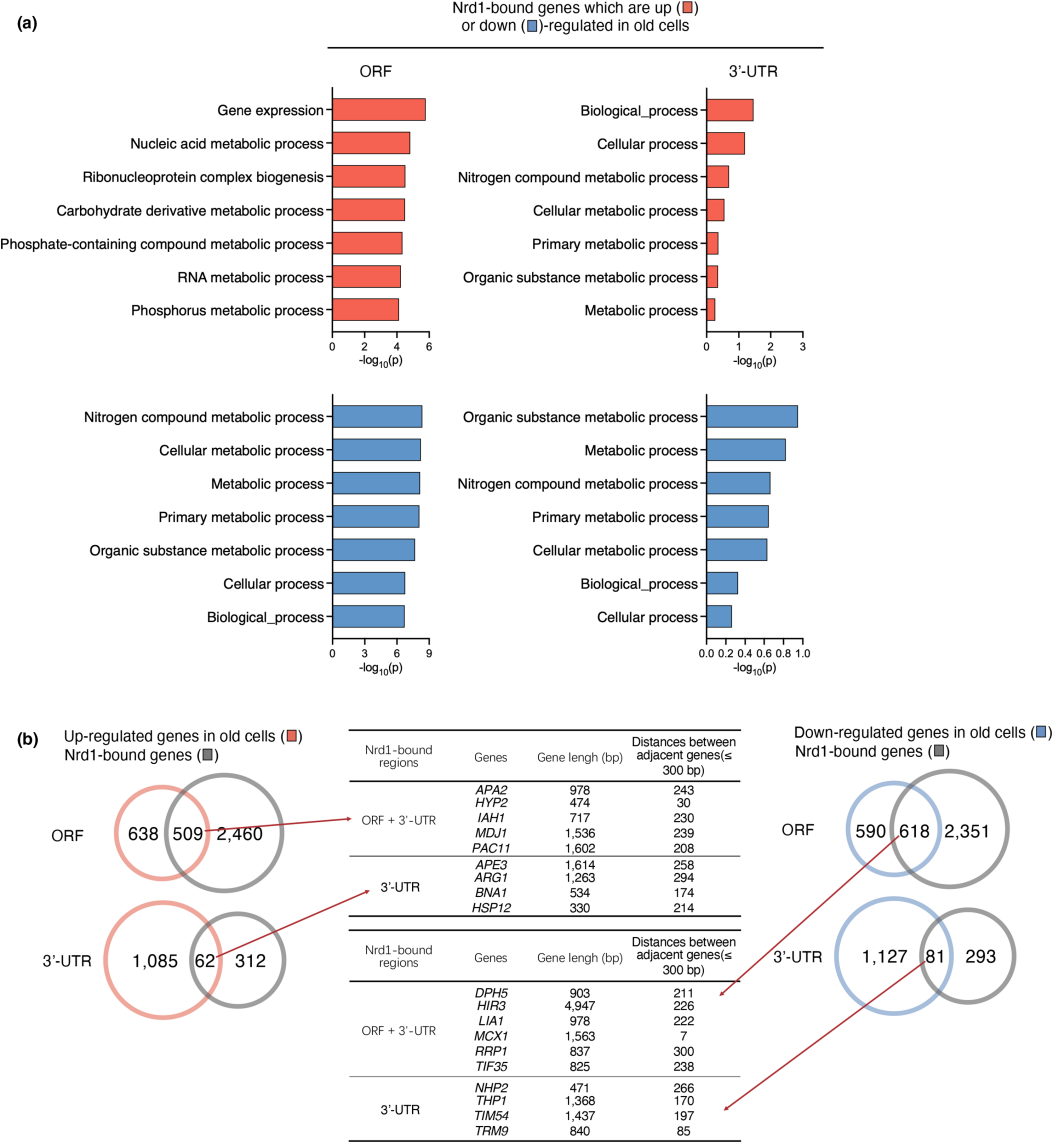
Comparative analysis of Nrd1‐bound genes with differentially expressed genes in aged cells. (a) GO analysis of Nrd1‐bound genes overlapped with up (red)‐ or down (blue)‐regulated genes in old cells, listed in Figure 6b. Bar diagrams indicate the fold‐enrichment of top seven GO biological process of Nrd1‐bound genes with binding sites in open reading frame (ORF) or 3′‐untranslated region (UTR). (b) Venn diagram of Nrd1‐bound genes displayed in (a). Middle tables show representative genes that Nrd1 binds to at both the ORF and 3′‐UTR or only the 3′‐UTR, and their length and distance from adjacent genes.

**FIGURE 8:**
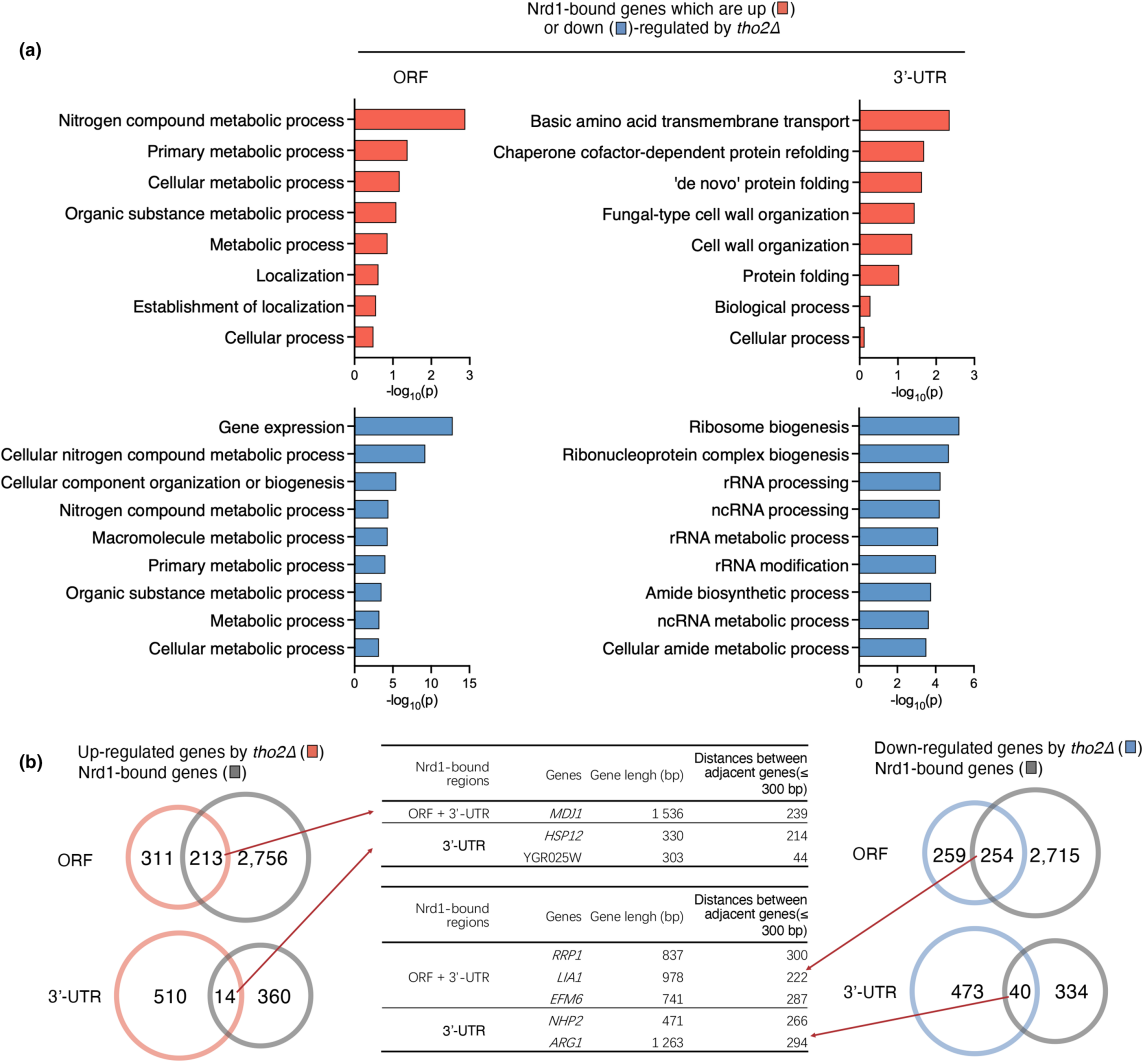
Comparison of Nrd1‐bound genes with differentially expressed genes in *tho2Δ* cells. (a) Top eight to nine GO analysis terms of Nrd1‐bound genes whose expression is up (red)‐ or down (blue)‐regulated by *tho2Δ*, as described in Figure 7a. (b) Venn diagram analysis of Nrd1‐bound genes displayed in (a), as described in Figure 7b.

Because the Nrd1 transcription termination factor plays a crucial role as a sequence‐specific RNA binding protein in yeast, participating in the processing and degradation of various RNA classes, we next compared the target RNAs of Nrd1 with differentially expressed genes in aged cells (Figure 7) and *tho2Δ* strains (Figure 8). In *S*.*cerevisiae*, almost all of mRNA sequences include the short UGUA and GUAG RNA motifs for Nrd1 binding. Nrd1 preferentially binds near the 5′ ends of relatively short transcripts, although the binding targets also include ORFs and 3′ untranslated regions (UTRs) of several hundred mRNAs (Webb et al., 2014). Therefore, we analyzed the loci of ORFs and 3′ UTRs of Nrd1‐bound mRNAs to avoid missing crucial information and to gain a more comprehensive understanding of cellular processes. Genes with Nrd1–3′ UTR binding did not exhibit substantial alterations in gene expression in aged cells (Figure 7b) or in *tho2Δ* mutants (Figure 8b). In both aged cells and *tho2Δ* mutants, Nrd1‐targeted genes were involved in gene expression, metabolism, and other functions (Figures 7a and 8a). In particular, Nrd1's 3’ UTR target mRNAs that show decreased expression in the *tho2Δ* strain were mainly associated with ribosome biogenesis, rRNA processing, and ncRNA processing. The Nrd1‐dependent transcription termination pathway is also linked to downregulation of mRNA levels by binding to 3′ UTRs (Webb et al., 2014). Unlike in the *tho2Δ* strain, translation‐associated genes were not primarily identified as Nrd1 targets in aging cells (Figures 7a and 8a), implying that not only aberrant expression of translation‐related genes but also multiple factors are involved in the aging. Furthermore, besides Nrd1, other factors may contribute to regulating the expression of genes related to translation in aging cells. Therefore, our data suggested that the translation or RNA processing genes whose expression was downregulated in the *tho2Δ* strain may be controlled through the Nrd1‐dependent pathway of gene expression regulation.

## 4 DISCUSSION

Herein, we revealed that Tho2, a THO complex subunit, is a novel regulatory factor in yeast lifespan regulation. The THO complex links transcription with mRNA export by coordinating interaction between diverse machineries such as the SAGA and TREX‐2 complexes (Garcia‐Oliver et al., 2012). While preventing abnormal accumulation of nuclear RNA is necessary for longevity (Lim et al., 2022; Park et al., 2022), the THO complex regulates lifespan independently of the Rrp6‐mediated RNA surveillance that ensures the quality of mRNA transcripts from impaired mRNA export. Instead of this, the interaction between the Nrd1 transcription termination factor and RNA polymerase II is indispensable for the THO complex‐dependent regulation of lifespan. The THO complex ensures a normal cellular lifespan by preventing the improper recruitment of Nrd1 to chromatin. Therefore, the Tho2‐facilitated Nrd1 recruitment is crucial for regulating the expression of genes essential for maintaining a typical lifespan.

The alterations in gene expression observed in *tho2Δ* cells were similar with those found in aged cells, particularly in genes associated with translation and RNA processing. This observation suggests a connection between the molecular pathways governed by the THO complex and the broader context of cellular aging. Moreover, Nrd1, whose recruitment is restricted by the THO complex, targets various genes that the THO complex also regulates, such as those involved in ribosome biogenesis, rRNA processing, and ncRNA processing. Nevertheless, since the mechanisms underlying how Nrd1 modulates RNA transcript levels remain unclear (Webb et al., 2014), further research in this area is warranted. However, our model illuminates the intersection of aging‐related processes with the functions of the THO complex and Nrd1, ensuring precise gene expression patterns important for a normal lifespan.

RNA metabolism and epigenetics are intricately interconnected processes, and the THO complex and Nrd1 act as critical enzymes within RNA metabolism. The THO complex collaborates with the SAGA complex, whose subunits are responsible for histone acetylation and H2B deubiquitination (Garcia‐Oliver et al., 2012). H3K4 trimethylation can facilitate the Nrd1‐mediated transcription termination pathway (Terzi et al., 2011). However, a broader array of histone modifications, such as sumoylation, acylation, or phosphorylation, exists beyond H3K4 methylation and histone acetylation (Chou et al., 2023; Ryu & Hochstrasser, 2021). A complex network of interactions is formed by the intricate interplay among these modifications. Therefore, a comprehensive investigation that explores these factors and their relationships with unexplored histone modifications is essential for a more complete understanding of their roles in epigenetics.

Overcoming aging has been a longstanding human aspiration. The intricacies of the causes and consequences of aging make research in this field challenging. In particular, the marked chromatin instability observed in aged cells drives alterations in gene expression, a process with critical implications for the aging process. Therefore, to understand how to prevent pathophysiological cell senescence and cell death, it is important to identify mechanisms that inhibit transcription errors, nuclear accumulation of RNA, and abnormal export of mature RNA. The freshly identified aging‐associated factor, THO complex, is conserved among higher eukaryotes and appears crucial for maintaining gene expression associated with aging.

## AUTHOR CONTRIBUTIONS

S.H.A. and H.‐Y.R. organized and designed the scope of the study. H.‐Y.R. wrote the manuscript with assistance from Y.L. and S.H.A. Y.L., S.L., D.Y.L., R.D., and B.‐H.R. performed the strain construction used in this study, spotting assay and/or lifespan analysis. S.L. performed RNA‐seq and data analysis. J.‐M.P., D.C., and D.K.C. performed ChIP‐seq and data anlaysis. S.Y.C. carried out re‐analysis of Nrd1‐bound genes. Y.L. and S.H.A. performed data analysis for all experiments.

## FUNDING INFORMATION

This study was supported by the National Research Foundation of Korea (NRF) grant funded by the South Korea government (MSIT) [RS‐2023‐00243165 to S.H.A.; RS‐2023‐00212894 to H.‐Y.R.], Learning & Academic research institution for Master's·PhD students, and Postdocs (LAMP) Program of the National Research Foundation of Korea (NRF) grant funded by the Ministry of Education [RS‐2023‐00301914 to H.‐Y.R.], and Korea Institute for Advancement of Technology funded by the Ministry of Trade, Industry and Energy [P0025489 to H.‐Y.R.]

## CONFLICT OF INTEREST STATEMENT

The authors declare no conflict of interest.

## Supporting information


Data S1.

**FIGURE S1.** *Δ*
**Figure S2.** *Δ*
Figure S3.

## Data Availability

Data generated in this publication have been deposited in Gene Expression Omnibus (GEO) with accession numbers:• RNA‐seq data: GSE262156 (token: ehiveyikrzydzcl)• ChIP‐seq data: GSE261087 (token: gbidqmoyztqbrqr).

## References

[acel14203-bib-0001] Camblong, J. , Iglesias, N. , Fickentscher, C. , Dieppois, G. , & Stutz, F. (2007). Antisense RNA stabilization induces transcriptional gene silencing via histone deacetylation in s. cerevisiae. Cell, 131(4), 706–717. 10.1016/j.cell.2007.09.014 18022365

[acel14203-bib-0002] Cho, U. H. , & Hetzer, M. W. (2020). Nuclear periphery takes center stage: The role of nuclear pore complexes in cell identity and aging. Neuron, 106(6), 899–911. 10.1016/j.neuron.2020.05.031 32553207 PMC9041311

[acel14203-bib-0003] Choi, J. , Ryoo, Z. Y. , Cho, D. H. , Lee, H. S. , & Ryu, H. Y. (2021). Trans‐tail regulation‐mediated suppression of cryptic transcription. Experimental & Molecular Medicine, 53(11), 1683–1688. 10.1038/s12276-021-00711-x 34845331 PMC8639711

[acel14203-bib-0004] Chou, K. Y. , Lee, J. Y. , Kim, K. B. , Kim, E. , Lee, H. S. , & Ryu, H. Y. (2023). Histone modification in Saccharomyces cerevisiae: A review of the current status. Computational and Structural Biotechnology Journal, 21, 1843–1850. 10.1016/j.csbj.2023.02.037 36915383 PMC10006725

[acel14203-bib-0005] Conrad, N. K. , Wilson, S. M. , Steinmetz, E. J. , Patturajan, M. , Brow, D. A. , Swanson, M. S. , & Corden, J. L. (2000). A yeast heterogeneous nuclear ribonucleoprotein complex associated with RNA polymerase II. Genetics, 154(2), 557–571. 10655211 10.1093/genetics/154.2.557 PMC1460961

[acel14203-bib-0006] Dang, W. , Steffen, K. K. , Perry, R. , Dorsey, J. A. , Johnson, F. B. , Shilatifard, A. , Kaeberlein, M. , Kennedy, B. K. , & Berger, S. L. (2009). Histone H4 lysine 16 acetylation regulates cellular lifespan. Nature, 459(7248), 802–807. 19516333 10.1038/nature08085 PMC2702157

[acel14203-bib-0007] Fasken, M. B. , Laribee, R. N. , & Corbett, A. H. (2015). Nab3 facilitates the function of the TRAMP complex in RNA processing via recruitment of Rrp6 independent of Nrd1. PLoS Genetics, 11(3), e1005044. 10.1371/journal.pgen.1005044 25775092 PMC4361618

[acel14203-bib-0008] Fontana, L. , Partridge, L. , & Longo, V. D. (2010). Extending healthy life span—From yeast to humans. Science, 328(5976), 321–326. 20395504 10.1126/science.1172539 PMC3607354

[acel14203-bib-0009] Fox, M. J. , & Mosley, A. L. (2016). Rrp6: Integrated roles in nuclear RNA metabolism and transcription termination. Wiley Interdisciplinary Reviews: RNA, 7(1), 91–104. 10.1002/wrna.1317 26612606 PMC4715707

[acel14203-bib-0010] Garcia‐Oliver, E. , Garcia‐Molinero, V. , & Rodriguez‐Navarro, S. (2012). mRNA export and gene expression: The SAGA‐TREX‐2 connection. Biochimica et Biophysica Acta, 1819(6), 555–565. 10.1016/j.bbagrm.2011.11.011 22178374

[acel14203-bib-0011] Gudipati, R. K. , Villa, T. , Boulay, J. , & Libri, D. (2008). Phosphorylation of the RNA polymerase II C‐terminal domain dictates transcription termination choice. Nature Structural & Molecular Biology, 15(8), 786–794. 10.1038/nsmb.1460 18660821

[acel14203-bib-0012] Hendrickson, D. G. , Soifer, I. , Wranik, B. J. , Kim, G. , Robles, M. , Gibney, P. A. , & McIsaac, R. S. (2018). A new experimental platform facilitates assessment of the transcriptional and chromatin landscapes of aging yeast. eLife, 7, e39911. 10.7554/eLife.39911 30334737 PMC6261268

[acel14203-bib-0013] Heo, D. H. , Yoo, I. , Kong, J. , Lidschreiber, M. , Mayer, A. , Choi, B. Y. , Hahn, Y. , Cramer, P. , Buratowski, S. , & Kim, M. (2013). The RNA polymerase II C‐terminal domain‐interacting domain of yeast Nrd1 contributes to the choice of termination pathway and couples to RNA processing by the nuclear exosome. The Journal of Biological Chemistry, 288(51), 36676–36690. 10.1074/jbc.M113.508267 24196955 PMC3868778

[acel14203-bib-0014] Hieronymus, H. , Yu, M. C. , & Silver, P. A. (2004). Genome‐wide mRNA surveillance is coupled to mRNA export. Genes & Development, 18(21), 2652–2662. 10.1101/Gad.1241204 15489286 PMC525545

[acel14203-bib-0015] Honorine, R. , Mosrin‐Huaman, C. , Hervouet‐Coste, N. , Libri, D. , & Rahmouni, A. R. (2011). Nuclear mRNA quality control in yeast is mediated by Nrd1 co‐transcriptional recruitment, as revealed by the targeting of rho‐induced aberrant transcripts. Nucleic Acids Research, 39(7), 2809–2820. 21113025 10.1093/nar/gkq1192 PMC3074134

[acel14203-bib-0016] Jamonnak, N. , Creamer, T. J. , Darby, M. M. , Schaughency, P. , Wheelan, S. J. , & Corden, J. L. (2011). Yeast Nrd1, Nab3, and Sen1 transcriptome‐wide binding maps suggest multiple roles in post‐transcriptional RNA processing. RNA, 17(11), 2011–2025. 10.1261/rna.2840711 21954178 PMC3198594

[acel14203-bib-0017] Jang, H. , Jo, Y. , Lee, J. H. , & Choi, S. (2023). Aging of hair follicle stem cells and their niches. BMB Reports, 56(1), 2–9. 10.5483/BMBRep.2022-0183 36379515 PMC9887102

[acel14203-bib-0018] Janssens, G. E. , Meinema, A. C. , Gonzalez, J. , Wolters, J. C. , Schmidt, A. , Guryev, V. , Bischoff, R. , Wit, E. C. , Veenhoff, L. M. , & Heinemann, M. (2015). Protein biogenesis machinery is a driver of replicative aging in yeast. eLife, 4, e08527. 10.7554/eLife.08527 26422514 PMC4718733

[acel14203-bib-0019] Jimeno, S. , Rondon, A. G. , Luna, R. , & Aguilera, A. (2002). The yeast THO complex and mRNA export factors link RNA metabolism with transcription and genome instability. EMBO Journal, 21(13), 3526–3535. 10.1093/emboj/cdf335 12093753 PMC126085

[acel14203-bib-0020] Kaeberlein, M. , McVey, M. , & Guarente, L. (1999). The SIR2/3/4 complex and SIR2 alone promote longevity in Saccharomyces cerevisiae by two different mechanisms. Genes & Development, 13(19), 2570–2580. 10.1101/gad.13.19.2570 10521401 PMC317077

[acel14203-bib-0021] Kim, H. , Cho, B. , Moon, S. , & Chung, Y. D. (2011). The THO complex is required for stress tolerance and longevity in Drosophila. Genes & Genomics, 33(3), 291–297.

[acel14203-bib-0022] Lai, R. W. , Lu, R. , Danthi, P. S. , Bravo, J. I. , Goumba, A. , Sampathkumar, N. K. , & Benayoun, B. A. (2019). Multi‐level remodeling of transcriptional landscapes in aging and longevity. BMB Reports, 52(1), 86–108. 30526773 10.5483/BMBRep.2019.52.1.296 PMC6386224

[acel14203-bib-0023] Langmead, B. , Wilks, C. , Antonescu, V. , & Charles, R. (2019). Scaling read aligners to hundreds of threads on general‐purpose processors. Bioinformatics, 35(3), 421–432. 10.1093/bioinformatics/bty648 30020410 PMC6361242

[acel14203-bib-0024] Libri, D. , Dower, K. , Boulay, J. , Thomsen, R. , Rosbash, M. , & Jensen, T. H. (2002). Interactions between mRNA export commitment, 3'‐end quality control, and nuclear degradation. Molecular and Cellular Biology, 22(23), 8254–8266. 10.1128/Mcb.22.23.8254-8266.2002 12417728 PMC134070

[acel14203-bib-0025] Lim, S. , Ahn, H. , Duan, R. , Liu, Y. , Ryu, H. Y. , & Ahn, S. H. (2021). The Spt7 subunit of the SAGA complex is required for the regulation of lifespan in both dividing and nondividing yeast cells. Mechanisms of Ageing and Development, 196, 111480. 10.1016/j.mad.2021.111480 33831401

[acel14203-bib-0026] Lim, S. , Liu, Y. , Rhie, B. H. , Kim, C. , Ryu, H. Y. , & Ahn, S. H. (2022). Sus1 maintains a normal lifespan through regulation of TREX‐2 complex‐mediated mRNA export. Aging, 14(12), 4990–5012. 35771153 10.18632/aging.204146 PMC9271307

[acel14203-bib-0027] Longo, V. D. , Shadel, G. S. , Kaeberlein, M. , & Kennedy, B. (2012). Replicative and chronological aging in *Saccharomyces cerevisiae* . Cell Metabolism, 16(1), 18–31. 10.1016/j.cmet.2012.06.002 22768836 PMC3392685

[acel14203-bib-0028] Longtine, M. S. , McKenzie, A., 3rd , Demarini, D. J. , Shah, N. G. , Wach, A. , Brachat, A. , & Pringle, J. R. (1998). Additional modules for versatile and economical PCR‐based gene deletion and modification in *Saccharomyces cerevisiae* . Yeast, 14(10), 953–961. 10.1002/(SICI)1097-0061(199807)14:10<953::AID-YEA293>3.0.CO;2-U 9717241

[acel14203-bib-0029] Luna, R. , Jimeno, S. , Marin, M. , Huertas, P. , Garcia‐Rubio, M. , & Aguilera, A. (2005). Interdependence between transcription and mRNP processing and export, and its impact on genetic stability. Molecular Cell, 18(6), 711–722. 10.1016/j.molcel.2005.05.001 15949445

[acel14203-bib-0030] Martin, M. (2011). Cutadapt removes adapter sequences from high‐throughput sequencing reads. EMBnet.Journal, 17(1), 3. 10.14806/ej.17.1.200

[acel14203-bib-0031] Merker, R. J. , & Klein, H. L. (2002). hpr1*Δ* affects ribosomal DNA recombination and cell life span in Saccharomyces cerevisiae. Molecular and Cellular Biology, 22(2), 421–429. 11756539 10.1128/MCB.22.2.421-429.2002 PMC139738

[acel14203-bib-0032] Mullani, N. , Porozhan, Y. , Mangelinck, A. , Rachez, C. , Costallat, M. , Batsche, E. , Goodhardt, M. , Cenci, G. , Mann, C. , & Muchardt, C. (2021). Reduced RNA turnover as a driver of cellular senescence. Life Science Alliance, 4(3), e202000809. 10.26508/lsa.202000809 33446491 PMC7812316

[acel14203-bib-0033] Park, H. S. , Lee, J. , Lee, H. S. , Ahn, S. H. , & Ryu, H. Y. (2022). Nuclear mRNA export and aging. International Journal of Molecular Sciences, 23(10), 5351. 10.3390/ijms23105451 35628261 PMC9142925

[acel14203-bib-0034] Rehwinkel, J. , Herold, A. , Gari, K. , Kocher, T. , Rode, M. , Ciccarelli, F. L. , Wilm, M. , & Izaurralde, E. (2004). Genome‐wide analysis of mRNAs regulated by the THO complex in Drosophila melanogaster. Nature Structural & Molecular Biology, 11(6), 558–566. 10.1038/nsmb759 15133499

[acel14203-bib-0035] Rempel, I. L. , Crane, M. M. , Thaller, D. J. , Mishra, A. , Jansen, D. P. , Janssens, G. , Popken, P. , Akşit, A. , Kaeberlein, M. , van der Giessen, E. , Steen, A. , Onck, P. R. , Lusk, C. P. , & Veenhoff, L. M. (2019). Age‐dependent deterioration of nuclear pore assembly in mitotic cells decreases transport dynamics. eLife, 8, e48186. 10.7554/eLife.48186 31157618 PMC6579512

[acel14203-bib-0036] Rodriguez‐Navarro, S. (2009). Insights into SAGA function during gene expression. EMBO Reports, 10(8), 843–850. 10.1038/embor.2009.168 19609321 PMC2726681

[acel14203-bib-0037] Ryu, H. Y. (2022). SUMO pathway is required for ribosome biogenesis. BMB Reports, 55(11), 535–540. 10.5483/BMBRep.2022.55.11.130 36195568 PMC9712707

[acel14203-bib-0038] Ryu, H. Y. , & Ahn, S. (2014). Yeast histone H3 lysine 4 demethylase Jhd2 regulates mitotic rDNA condensation. BMC Biology, 12, 75. 10.1186/s12915-014-0075-3 25248920 PMC4201760

[acel14203-bib-0039] Ryu, H. Y. , & Hochstrasser, M. (2021). Histone sumoylation and chromatin dynamics. Nucleic Acids Research, 49(11), 6043–6052. 10.1093/nar/gkab280 33885816 PMC8216275

[acel14203-bib-0040] Ryu, H. Y. , Lopez‐Giraldez, F. , Knight, J. , Hwang, S. S. , Renner, C. , Kreft, S. G. , & Hochstrasser, M. (2018). Distinct adaptive mechanisms drive recovery from aneuploidy caused by loss of the Ulp2 SUMO protease. Nature Communications, 9(1), 5417. 10.1038/s41467-018-07836-0 PMC6303320 30575729

[acel14203-bib-0041] Ryu, H.‐Y. , Rhie, B.‐H. , & Ahn, S. H. (2014). Loss of the Set2 histone methyltransferase increases cellular lifespan in yeast cells. Biochemical and Biophysical Research Communications, 446, 113–118. 24607280 10.1016/j.bbrc.2014.02.061

[acel14203-bib-0042] Ryu, H. Y. , Su, D. , Wilson‐Eisele, N. R. , Zhao, D. J. , Lopez‐Giraldez, F. , & Hochstrasser, M. (2019). The Ulp2 SUMO protease promotes transcription elongation through regulation of histone sumoylation. EMBO Journal, 38(16), e102003. 10.15252/embj.2019102003 31313851 PMC6694223

[acel14203-bib-0043] Ryu, H. Y. , Wilson, N. R. , Mehta, S. , Hwang, S. S. , & Hochstrasser, M. (2016). Loss of the SUMO protease Ulp2 triggers a specific multichromosome aneuploidy. Genes & Development, 30(16), 1881–1894. 10.1101/gad.282194.116 27585592 PMC5024685

[acel14203-bib-0044] Ryu, H. Y. , Zhao, D. , Li, J. , Su, D. , & Hochstrasser, M. (2020). Histone sumoylation promotes Set3 histone‐deacetylase complex‐mediated transcriptional regulation. Nucleic Acids Research, 48, 12151–12168. 10.1093/nar/gkaa1093 33231641 PMC7708062

[acel14203-bib-0045] Sinclair, D. A. , & Guarente, L. (1997). Extrachromosomal rDNA circles—A cause of aging in yeast. Cell, 91(7), 1033–1042. 9428525 10.1016/s0092-8674(00)80493-6

[acel14203-bib-0046] Son, H. G. , & Lee, S. V. (2017). Longevity regulation by NMD‐mediated mRNA quality control. BMB Reports, 50(4), 160–161. 10.5483/bmbrep.2017.50.4.045 28288699 PMC5437958

[acel14203-bib-0047] Strambio‐De‐Castillia, C. , Niepel, M. , & Rout, M. P. (2010). The nuclear pore complex: Bridging nuclear transport and gene regulation. Nature Reviews Molecular Cell Biology, 11(7), 490–501. 10.1038/nrm2928 20571586

[acel14203-bib-0048] Strasser, K. , Masuda, S. , Mason, P. , Pfannstiel, J. , Oppizzi, M. , Rodriguez‐Navarro, S. , Rondón, A. G. , Aguilera, A. , Struhl, K. , Reed, R. , & Hurt, E. (2002). TREX is a conserved complex coupling transcription with messenger RNA export. Nature, 417(6886), 304–308. 10.1038/nature746 11979277

[acel14203-bib-0049] Terzi, N. , Churchman, L. S. , Vasiljeva, L. , Weissman, J. , & Buratowski, S. (2011). H3K4 trimethylation by Set1 promotes efficient termination by the Nrd1‐Nab3‐Sen1 pathway. Molecular and Cellular Biology, 31(17), 3569–3583. 10.1128/MCB.05590-11 21709022 PMC3165552

[acel14203-bib-0050] Vasiljeva, L. , Kim, M. , Mutschler, H. , Buratowski, S. , & Meinhart, A. (2008). The Nrd1‐Nab3‐Sen1 termination complex interacts with the Ser5‐phosphorylated RNA polymerase II C‐terminal domain. Nature Structural & Molecular Biology, 15(8), 795–804. 10.1038/nsmb.1468 PMC2597375 18660819

[acel14203-bib-0051] Vinciguerra, P. , & Stutz, F. (2004). MRNA export: An assembly line from genes to nuclear pores. Current Opinion in Cell Biology, 16(3), 285–292. 10.1016/j.ceb.2004.03.013 15145353

[acel14203-bib-0052] Webb, S. , Hector, R. D. , Kudla, G. , & Granneman, S. (2014). PAR‐CLIP data indicate that Nrd1‐Nab3‐dependent transcription termination regulates expression of hundreds of protein coding genes in yeast. Genome Biology, 15(1) R8, R8. 10.1186/gb-2014-15-1-r8 24393166 PMC4053934

[acel14203-bib-0053] Whang, J. , Ahn, C. , Kim, S. , Seok, E. , Yang, Y. , Han, G. , Jo, H. , & Yang, H. (2021). Effects of repeated ovarian stimulation on ovarian function and aging in mice. Development & Reproduction, 25(4), 213–223. 10.12717/DR.2021.25.4.213 35141447 PMC8807135

[acel14203-bib-0054] Zhang, D. W. , Rodriguez‐Molina, J. B. , Tietjen, J. R. , Nemec, C. M. , & Ansari, A. Z. (2012). Emerging views on the CTD code. Genetics Research International, 2012, 1–19. 10.1155/2012/347214 PMC3335543 22567385

