# Supplementary material for: Tho2‐mediated escort of Nrd1 regulates the expression of aging‐related genes
Source: Aging Cell. 2024 May 20;23(8):e14203. doi: 10.1111/acel.14203 (PMC11320360; doi:10.1111/acel.14203)
Supplement: Supplementary file 1 — Data S1. [file ACEL-23-e14203-s002.docx]

**Supplementary Figure legends**

**FIGURE S1.** RLS analysis of exosome components. (a-c) RLS analysis of WT and indicated mutants, as described in **Figure 1**. pCM189 or pCM189-Rho were transformed to WT or *trf4Δ* (b) or *sen1-1* (c). Growth analysis of strains in (b) and (c) was performed on Rho-repressing (+Doxy) or Rho-inducing (−Doxy) plates, as described in **Figure 2a**.

**FIGURE S2.** RLS analysis of WT or *hpr1Δ* strains transformed with empty vector (pAG425GPD) or plasmid expressing Nrd1 1-151 truncate, as described in **Figure 1**.

**FIGURE S3.** RLS analysis of *nrd1* mutants. (a, b) RLS analysis of WT and the indicated mutants, as described in **Figure 1**.

**Table S1.** Strains used in this study.

| Strain | Genotype | Source |
| --- | --- | --- |
| BY4741 | *MATa ura3∆0 leu2∆0 his3∆1 met15∆0* | EUROSCARF |
| FY164 | *MATa ura3Δ0 leu2Δ0 his3Δ1 met15Δ0 hpr1Δ*::*KanMX4* | EUROSCARF |
| FY252 | *MATa ura3Δ0 leu2Δ0 his3Δ1 met15Δ0 rrp6Δ*::*KanMX4* | EUROSCARF |
| FY254 | *MATa ura3Δ0 leu2Δ0 his3Δ1 met15Δ0 tho2Δ*::*KanMX4* | EUROSCARF |
| FY286 (W303α) | *MATα ura3-1 leu2-3,112 trp1-1 his3-11,15 ade2-1* | François Lacroute (Minvielle-Sebastia, Winsor, Bonneaud, & Lacroute, 1991) |
| FY364 | *MATa ura3Δ0 leu2Δ0 his3Δ1 met15Δ0 cyh2 rna14*-DAmP::Kan^R^ | Open Biosystems |
| FY369 (YJC610) | *MATa ura3Δ0 leu2Δ0 his3Δ1 met15Δ0 nrd1Δ*::*KAN* [pJC580] | Jeffry L. Corden (Conrad et al., 2000) |
| FY370 (YJC818) | *MATa ura3Δ0 leu2Δ0 his3Δ1 met15Δ0 nrd1Δ*::*KAN* [pJC951] | Jeffry L. Corden (Conrad et al., 2000) |
| FY371 (YJC1107) | *MATa ura3Δ0 leu2Δ0 his3Δ1 met15Δ0 nrd1Δ*::*KAN* [pJC720] | Jeffry L. Corden (Conrad et al., 2000) |
| FY378 | *MATa ura3Δ0 leu2Δ0 his3Δ1 met15Δ0 cyh2 pcf11*-DAmP::Kan^R^ | Open Biosystems |
| FY380 | *MATa ura3Δ0 leu2Δ0 his3Δ1 met15Δ0 mft1Δ*::*KanMX4* | EUROSCARF |
| FY381 | *MATa ura3Δ0 leu2Δ0 his3Δ1 met15Δ0 thp2Δ*::*KanMX4* | EUROSCARF |
| FY393 | *MATa ura3Δ0 leu2Δ0 his3Δ1 met15Δ0 tex1Δ*::*KanMX4* | EUROSCARF |
| FY455 | *MATa ura3Δ0 leu2Δ0 his3Δ1 met15Δ0 xrn1Δ*::*KanMX4* | EUROSCARF |
| FY456 | *MATa ura3Δ0 leu2Δ0 his3Δ1 met15Δ0 ski2Δ*::*KanMX4* | EUROSCARF |
| FY520 (DLY157) | *MATα ura3-1 leu2-3,112 trp1-1 his3-11,15 ade2-1 can1-100 hpr1Δ*::*HIS3MX6* | Domenico Libri (Libri et al., 2002) |
| FY522 (DLY190) | *MATα ura3-1 leu2-3,112 trp1-1 his3-11,15 ade2-1 can1-100 hpr1Δ*::*HIS3MX6 rrp6Δ*::*KanMX4* | Domenico Libri (Libri et al., 2002) |
| FY556 (Y246) | *MATa ura3-1 leu2-3,112 trp1-1 his3-11,15 ade2-1 can1-100 tho2Δ*::*KAN* | Andrés Aguilera (Piruat & Aguilera, 1998) |
| FY557 (Y1928) | *MATα ura3-1 leu2-3,112 trp1-1 his3-11,15 ade2-1 can1-100 pap1-1* | J. Scott Butler (Patel & Butler, 1992) |
| FY558 (Y1929) | *MATα ura3-52 leu2-3,112 trp1 his4 fip1Δ*::*LEU2* [pTRP1-fip1-206] | Claire L. Moore (Helmling, Zhelkovsky, & Moore, 2001) |
| FY559 (Y1913) | *MATα ura3-1 leu2-3,112 trp1-1 his3-11,15 ade2-1 can1-100 tho2Δ*::*KAN pap1-1* | Torben Heick Jensen (Saguez et al., 2008) |
| FY560 (Y2033) | *MATα ura3-52 leu2-3,112 trp1 his4 fip1Δ*::*LEU2 tho2Δ*::*KAN* [pTRP1-fip1-216] | Torben Heick Jensen (Saguez et al., 2008) |
| FY562 (YF2362 / SH1A) | *MATα ura3-52 leu2-3,112 trp1 his4 fip1Δ*::*LEU2* [p314Fip1] | Claire L. Moore (Helmling et al., 2001) |
| FY567 (YF1165 / EJS101-9d) | *MATa ura3-52 leu2-3,112 trp1-1 his3-11,15 nrd1Δ*::*HIS3 lys2Δ2 ade2-1 met2Δ1 can1-100* [pRS316-NRD1] | Eric J. Steinmetz / David A. Brow (Steinmetz & Brow, 1996) |
| FY568 (YSB1752 / nrd1-1) | *MATa ura3-52 leu2-3,112 trp1-1 his3-11,15 nrd1Δ*::*HIS3 lys2Δ2 ade2-1 met2Δ1 can1-100* [pRS424-nrd1-1] | Stephen Buratowski (Vasiljeva & Buratowski, 2006) |
| FY569 (YSB1753/ nrd1-2) | *MATa ura3-52 leu2-3,112 trp1-1 his3-11,15 nrd1Δ*::*HIS3 lys2Δ2 ade2-1 met2Δ1 can1-100* [pRS424-nrd1-2] | Stephen Buratowski (Vasiljeva & Buratowski, 2006) |
| FY570 (YSB1754 / nrd1Δ39-169) | *MATa ura3-52 leu2-3,112 trp1-1 his3-11,15 nrd1Δ*::*HIS3 lys2Δ2 ade2-1 met2Δ1 can1-100* [pRS314NRD1Δ39-169] | Stephen Buratowski (Vasiljeva & Buratowski, 2006) |
| SY529 | *MATa ura3Δ0 leu2Δ0 his3Δ1 met15Δ0 sir2Δ*::*HISMX6* | This study |
| SY531 | *MATa ura3Δ0 leu2Δ0 his3Δ1 met15Δ0 trf4Δ*::*HISMX6* | This study |
| SY560 | *MATa ura3Δ0 leu2Δ0 his3Δ1 met15Δ0 sir2Δ*::*HISMX6 hpr1Δ*::*KanMX4* | This study |
| SY561 | *MATa ura3Δ0 leu2Δ0 his3Δ1 met15Δ0 sir2Δ*::*HISMX6 tho2Δ*::*KanMX4* | This study |
| SY655 | *MATa ura3Δ0 leu2Δ0 his3Δ1 met15Δ0* [pCM189] | This study |
| SY656 | *MATa ura3Δ0 leu2Δ0 his3Δ1 met15Δ0* [pCM189-Rho] | This study |
| SY658 | *MATa ura3Δ0 leu2Δ0 his3Δ1 met15Δ0 rrp6Δ*::*KanMX4* [pCM189] | This study |
| SY659 | *MATa ura3Δ0 leu2Δ0 his3Δ1 met15Δ0 rrp6Δ*::*KanMX4* [pCM189-Rho] | This study |
| SY661 | *MATa ura3Δ0 leu2Δ0 his3Δ1 met15Δ0 trf4Δ*::*HISMX6* [pCM189] | This study |
| SY662 | *MATa ura3Δ0 leu2Δ0 his3Δ1 met15Δ0 trf4Δ*::*HISMX6* [pCM189-Rho] | This study |
| SY672 | *MATa ura3Δ0 leu2Δ0 his3Δ1 met15Δ0 nrd1Δ*::*KAN* [pJC580] [pCM189] | This study |
| SY673 | *MATa ura3Δ0 leu2Δ0 his3Δ1 met15Δ0 nrd1Δ*::*KAN* [pJC580] [pCM189-Rho] | This study |
| SY674 | *MATa ura3Δ0 leu2Δ0 his3Δ1 met15Δ0 nrd1Δ*::*KAN* [pJC951] [pCM189] | This study |
| SY675 | *MATa ura3Δ0 leu2Δ0 his3Δ1 met15Δ0 nrd1Δ*::*KAN* [pJC951] [pCM189-Rho] | This study |
| SY676 | *MATa ura3-52 leu2-3,112 trp1- pep4-3* [pCM189] | This study |
| SY677 | *MATa ura3-52 leu2-3,112 trp1- pep4-3* [pCM189-Rho] | This study |
| SY678 | *MATa ura3-52 leu2-3,112 trp1- pep4-3 sen1-1* [pCM189] | This study |
| SY679 | *MATa ura3-52 leu2-3,112 trp1- pep4-3 sen1-1* [pCM189-Rho] | This study |
| SY680 | *MATa ura3Δ0 leu2Δ0 his3Δ1 met15Δ0 rrp6Δ*::*HIS3MX6* | This study |
| SY724 | *MATa ura3Δ0 leu2Δ0 his3Δ1 met15Δ0 nrd1Δ*::*KAN tho2Δ*::*HIS3MX6* [pJC580] | This study |
| SY725 | *MATa ura3Δ0 leu2Δ0 his3Δ1 met15Δ0 nrd1Δ*::*KAN tho2Δ*::*HIS3MX6* [pJC951] | This study |
| SY761 | *MATa ura3Δ0 leu2Δ0 his3Δ1 met15Δ0 hpr1Δ*::*KanMX4 rrp6Δ*::*HIS3MX6* | This study |
| SY908 | *MATa ura3Δ0 leu2Δ0 his3Δ1 met15Δ0 nrd1Δ*::*KAN* [pJC720] [pCM189] | This study |
| SY909 | *MATa ura3Δ0 leu2Δ0 his3Δ1 met15Δ0 nrd1Δ*::*KAN* [pJC720] [pCM189-Rho] | This study |
| SY910 | *MATa ura3Δ0 leu2Δ0 his3Δ1 met15Δ0 nrd1Δ*::*KAN tho2Δ*::HIS3MX6 [pJC720] | This study |
| SY913 | *MATa ura3Δ0 leu2Δ0 his3Δ1 met15Δ0* [pAG425GPD] | This study |
| SY923 | *MATa ura3Δ0 leu2Δ0 his3Δ1 met15Δ0 tho2Δ*::*KanMX4* [pAG425GPD] | This study |
| SY942 | *MATa ura3Δ0 leu2Δ0 his3Δ1 met15Δ0 tho2Δ*::*KanMX4* [pAG425GPD-NRD1(1-151)] | This study |
| SY943 | *MATa ura3Δ0 leu2Δ0 his3Δ1 met15Δ0 tho2Δ*::*KanMX4* [pAG425GPD-NRD1(1-178)] | This study |
| SY944 | *MATa ura3Δ0 leu2Δ0 his3Δ1 met15Δ0 tho2Δ*::*KanMX4* [pAG425GPD-NRD1(1-369)] | This study |
| SY945 | *MATa ura3Δ0 leu2Δ0 his3Δ1 met15Δ0 tho2Δ*::*KanMX4* [pAG425GPD-NRD1(1-448)] | This study |
| SY946 | *MATa ura3Δ0 leu2Δ0 his3Δ1 met15Δ0 tho2Δ*::*KanMX4* [pAG425GPD-NRD1(ΔRRM)] | This study |
| SY950 | *MATa ura3-52 leu2-3,112 trp1-1 his3-11,15 nrd1Δ*::*HIS3 lys2Δ2 ade2-1 met2Δ1 can1-100 tho2Δ*::*KanMX4* [pRS316-NRD1] | This study |
| SY952 | *MATa ura3-52 leu2-3,112 trp1-1 his3-11,15 nrd1*Δ::*HIS3 lys2Δ2 ade2-1 met2Δ1 can1-100 tho2Δ*::*KanMX4* [pRS314NRD1Δ39-169] | This study |
| SY1181 | *MATa ura3∆0 leu2∆0 his3∆1 met15∆0* [pAG425GPD-NRD1] | This study |
| SY1182 | *MATa ura3Δ0 leu2Δ0 his3Δ1 met15Δ0 tho2Δ*::*KanMX4* [pAG425GPD-NRD1] | This study |
| SY1258 | *MATa ura3Δ0 leu2Δ0 his3Δ1 met15Δ0 hpr1Δ*::*KanMX4* [pAG425GPD] | This study |
| SY1259 | *MATa ura3Δ0 leu2Δ0 his3Δ1 met15Δ0 hpr1Δ*::*KanMX4* [pAG425GPD-NRD1(1-151)] | This study |
| SY1260 | *MATa ura3Δ0 leu2Δ0 his3Δ1 met15Δ0 hpr1Δ*::*KanMX4* [pAG425GPD-NRD1(1-178)] | This study |
| SY1261 | *MATa ura3Δ0 leu2Δ0 his3Δ1 met15Δ0 hpr1Δ*::*KanMX4* [pAG425GPD-NRD1(1-369)] | This study |
| SY1265 | *MATa ura3Δ0 leu2Δ0 his3Δ1 met15Δ0 nrd1Δ*::*KAN hpr1Δ*::*HIS3MX6* [pJC580] | This study |
| SY1266 | *MATa ura3Δ0 leu2Δ0 his3Δ1 met15Δ0 nrd1Δ*::*KAN hpr1Δ*::HIS3MX6 [pJC720] | This study |
| HYS674 | *MATa ura3∆0 leu2∆0 his3∆1 met15∆0 NRD1-TAP*::HIS3MX6 | Horizon Discovery |
| HYS676 | *MATa ura3∆0 leu2∆0 his3∆1 met15∆0 tho2Δ::KanMX4 NRD1-TAP*::HIS3MX6 | This study |

**Table S2.** Plasmids used in this study.

| Name | Description | Source |
| --- | --- | --- |
| pCM189 | *CEN, Amp^R^, URA3* | A. Rachid Rahmouni (Honorine, Mosrin-Huaman, Hervouet-Coste, Libri, & Rahmouni, 2011) |
| pCM189-Rho | *CEN, Amp^R^, URA3, pTetO7::Rho-NLS* | A. Rachid Rahmouni (Honorine et al., 2011) |
| pAG425GPD | *2μ, Amp^R^, LEU2,* P*_GPD_* | A. Rachid Rahmouni (Honorine et al., 2011) |
| pAG425GPD-NRD1 | *2μ, Amp^R^, LEU2,* P*_GPD_, NRD1* | A. Rachid Rahmouni (Honorine et al., 2011) |
| pAG425GPD-NRD1(1-151) | *2μ, Amp^R^, LEU2,* P*_GPD_, NRD1(1-151)* | A. Rachid Rahmouni (Honorine et al., 2011) |
| pAG425GPD-NRD1(1-178) | *2μ, Amp^R^, LEU2,* P*_GPD_, NRD1(1-178)* | A. Rachid Rahmouni (Honorine et al., 2011) |
| pAG425GPD-NRD1(1-369) | *2μ, Amp^R^, LEU2,* P*_GPD_, NRD1(1-369)* | A. Rachid Rahmouni (Honorine et al., 2011) |
| pAG425GPD-NRD1(1-448) | *2μ, Amp^R^, LEU2,* P*_GPD_, NRD1(1-448)* | A. Rachid Rahmouni (Honorine et al., 2011) |
| pAG425GPD-NRD1(ΔRRM) | *2μ, Amp^R^, LEU2,* P*_GPD_, NRD1(Δ340-410)* | A. Rachid Rahmouni (Honorine et al., 2011) |

**Table S3.** Mean lifespans and *p*-values for RLS analysis.

| Figure | Strain | Strain description | Mean lifespan | *p*-value compared to matched control strain | *p*-value |
| --- | --- | --- | --- | --- | --- |
| Figure 1a | BY4741 | WT | 28.4 | control |  |
|  | FY164 | *hpr1Δ* | 3.5 | <0.0001, *** |  |
|  | FY254 | *tho2Δ* | 8.9 | <0.0001, *** |  |
|  | FY380 | *mft1Δ* | 26.0 | 0.3531, ns |  |
|  | FY381 | *thp2Δ* | 25.8 | 0.1984, ns |  |
|  | FY393 | *tex1Δ* | 25.9 | 0.1344, ns |  |
| Figure 1b | BY4741 | WT | 23.8 | control |  |
|  | SY529 | *sir2Δ* | 14.0 | <0.0001, *** |  |
|  | FY164 | *hpr1Δ* | 5.1 | <0.0001, *** |  |
|  | FY254 | *tho2Δ* | 14.5 | <0.0001, *** |  |
|  | SY560 | *sir2Δ hpr1Δ* | 3.6 | <0.0001, *** | <0.0001, ***  (compared to *sir2Δ*); 0.0158, *  (compared to *hpr1Δ*) |
|  | SY561 | *sir2Δ tho2Δ* | 4.1 | <0.0001, *** | <0.0001, ***  (compared to *sir2Δ*); <0.0001, ***  (compared to *tho2Δ*) |
| Figure 2a | SY655 | WT | 21.6 | control |  |
|  | SY656 | Rho | 17.5 | 0.0018, ** |  |
|  | SY658 | *rrp6Δ* | 14.7 | <0.0001, *** |  |
|  | SY659 | Rho + *rrp6Δ* | 27.6 | 0.0019, ** | <0.0001, ***  (compared to Rho); <0.0001, ***  (compared to *rrp6Δ*) |
| Figure 2b | BY4741 | WT (BY4741) | 23.0 | control |  |
|  | FY286 (W303α) | WT (W303α) | 21.3 | control |  |
|  | FY164 | *hpr1Δ* | 8.6 | <0.0001, *** (compared to BY4741) |  |
|  | FY520 (DLY157) | *hpr1Δ* (DLY157) | 9.2 | <0.0001, *** (compared to W303α) |  |
|  | SY761 | *hpr1Δ rrp6Δ* | 3.3 | <0.0001, *** (compared to BY4741) | <0.0001, *** (compared to *hpr1Δ*) |
|  | FY522 (DLY190) | *hpr1Δ rrp6Δ* (DLY190) | 2.8 | <0.0001, *** (compared to W303α) | <0.0001, *** (compared to *hpr1Δ*) |
| Figure 3c | FY369 | WT | 17.4 | control |  |
|  | FY370 | *nrd1-51* | 13.2 | 0.0375, * |  |
|  | FY371 | *nrd1-102* | 18.1 | 0.5626, ns |  |
| Figure 3d | SY672 | WT | 20.7 | control |  |
|  | SY673 | Rho | 17.2 | 0.0480, * |  |
|  | SY674 | *nrd1-51* | 14.0 | <0.0001, *** |  |
|  | SY675 | Rho + *nrd1-51* | 23.6 | 0.0850, ns | <0.0001, *** (compared to Rho)  <0.0001, ***  (compared to *nrd1-51*) |
| Figure 3e | SY672 | WT | 19.6 | control |  |
|  | SY673 | Rho | 14.1 | 0.0166, * |  |
|  | SY908 | *nrd1-102* | 18.4 | 0.6377, ns |  |
|  | SY909 | Rho + *nrd1-102* | 18.0 | 0.5058, ns | 0.0588, ns  (compared to Rho); 0.8946, ns  (compared to *nrd1-102*) |
| Figure 4b | FY369 | WT | 20.4 | control |  |
|  | SY1265 | *hpr1Δ* | 7.1 | <0.0001, *** |  |
|  | FY371 | *nrd1-102* | 14.0 | 0.0045, ** |  |
|  | SY1266 | *hpr1Δ + nrd1-102* | 13.8 | 0.0030, ** | <0.0001, ***  (compared to *hpr1Δ*); 0.8639, ns  (compared to *nrd1-102*) |
| Figure 4c | FY369 | WT | 16.9 | control |  |
|  | SY724 | *tho2Δ* | 3.6 | <0.0001, *** |  |
|  | FY371 | *nrd1-102* | 10.7 | 0.0019, ** |  |
|  | SY910 | *tho2Δ + nrd1-102* | 11.2 | 0.0065, ** | <0.0001, ***  (compared to *tho2Δ*); 0.7011, ns  (compared to *nrd1-102*) |
| Figure 4d | SY913 | WT | 24.6 | control |  |
|  | SY923 | *tho2Δ* | 11.3 | <0.0001, *** |  |
|  | SY942 | *tho2Δ +* Nrd1 1-151 | 16.9 | <0.0001, *** | <0.0001, ***  (compared to *tho2Δ*) |
|  | SY943 | *tho2Δ +* Nrd1 1-178 | 14.8 | <0.0001, *** | 0.0280, *  (compared to *tho2Δ*) |
|  | SY944 | *tho2Δ +* Nrd1 1-369 | 20.6 | 0.0387, * | <0.0001, ***  (compared to *tho2Δ*) |
|  | SY945 | *tho2Δ +* Nrd1 1-448 | 20.5 | 0.0441, * | <0.0001, ***  (compared to *tho2Δ*) |
|  | SY946 | *tho2Δ +* Nrd1 ΔRRM | 15.7 | <0.0001, *** | 0.0072, **  (compared to *tho2Δ*) |
| Figure 4e | FY567 | *nrd1Δ NRD1* | 11.7 | control |  |
|  | FY568 | *nrd1Δ nrd1-1* | 5.7 | 0.0012, ** |  |
|  | FY569 | *nrd1Δ nrd1-2* | 3.9 | <0.0001, *** |  |
|  | FY570 | *nrd1Δ nrd1ΔCID* | 5.1 | <0.0001, *** |  |
| Figure 4f | FY369 | WT | 20.6 | control |  |
|  | SY724 | *tho2Δ* | 4.8 | <0.0001, *** |  |
|  | FY370 | *nrd1-51* | 18.2 | 0.3224, ns |  |
|  | SY725 | *tho2Δ + nrd1-51* | 4.1 | <0.0001, *** | 0.3325, ns  (compared to *tho2Δ*); <0.0001, ***  (compared to *nrd1-51*) |
| Figure 4h | FY567 | *nrd1Δ NRD1* | 13.4 | control |  |
|  | SY950 | *tho2Δ nrd1Δ NRD1* | 4.9 | <0.0001, *** |  |
|  | SY952 | *tho2Δ nrd1Δ nrd1ΔCID* | 6.2 | <0.0001, *** | 0.0781, ns (compared to *tho2Δ nrd1Δ NRD1*) |
| Figure 5c | SY913 | WT | 13.1 | control |  |
|  | SY923 | *tho2Δ* | 3.9 | <0.0001, *** |  |
|  | SY1181 | NRD1-OE | 4.9 | <0.0001, *** | 0.8390, ns  (compared to *tho2Δ*) |
|  | SY1182 | *tho2Δ +* NRD1-OE | 4.0 | <0.0001, *** | 0.2588, ns  (compared to *tho2Δ*) |
| Figure S1a | BY4741 | WT | 29.7 | control |  |
|  | FY252 | *rrp6Δ* | 23.1 | 0.0011, ** |  |
|  | SY531 | *trf4Δ* | 10.4 | <0.0001, *** |  |
|  | FY455 | *xrn1Δ* | 14.8 | <0.0001, *** |  |
|  | FY456 | *ski2Δ* | 27.9 | 0.2638, ns |  |
| Figure S1b | SY655 | WT | 23.9 | control |  |
|  | SY656 | Rho | 16.9 | <0.0001, *** |  |
|  | SY661 | *trf4Δ* | 10.6 | <0.0001, *** |  |
|  | SY662 | Rho + *trf4Δ* | 14.0 | <0.0001, *** | 0.0350, *  (compared to Rho); 0.0364, *  (compared to *trf4Δ*) |
| Figure S1c | SY676 | WT | 28.9 | control |  |
|  | SY677 | Rho | 25.0 | 0.1255, ns |  |
|  | SY678 | *sen1-1* | 11.4 | <0.0001, *** |  |
|  | SY679 | Rho + *sen1-1* | 13.0 | <0.0001, *** | <0.0001, ***  (compared to Rho); 0.2043, ns  (compared to *sen1-1*) |
| Figure S2 | SY913 | WT | 25.1 | control |  |
|  | SY1258 | *hpr1Δ* | 5.9 | <0.0001, *** |  |
|  | SY1259 | *hpr1Δ +* Nrd1 1-151 | 13.8 | <0.0001, *** | <0.0001, ***  (compared to *hpr1Δ*) |
|  | SY1260 | *hpr1Δ +* Nrd1 1-178 | 7.4 | <0.0001, *** | 0.0047, **  (compared to *hpr1Δ*) |
|  | SY1261 | *hpr1Δ +* Nrd1 1-369 | 7.5 | <0.0001, *** | 0.0076, **  (compared to *hpr1Δ*) |
| Figure S3a | FY562 (SH1A) | WT (SH1A) | 16.7 | control |  |
|  | FY558 (Y1929) | *fip1-206* (Y1929) | 3.4 | <0.0001, *** |  |
|  | FY560 (Y2033) | *tho2Δ + fip1-216* (Y2033) | 4.2 | <0.0001, *** |  |
| Figure S3b | FY286 | WT | 13.3 | control |  |
|  | FY556 | *tho2Δ* | 2.5 | <0.0001, *** |  |
|  | FY557 | *pap1-1* | 7.4 | <0.0001, *** |  |
|  | FY559 | *tho2Δ + pap1-1* | 2.7 | <0.0001, *** | 0.2988, ns  (compared to *tho2Δ*); <0.0001, ***  (compared to *pap1-1*) |

***, *p* ≤ 0.0001; **, *p* ≤ 0.01; *, *p* ≤ 0.05; ns, not significant.

**Supplementary References**

Conrad, N. K., Wilson, S. M., Steinmetz, E. J., Patturajan, M., Brow, D. A., Swanson, M. S., & Corden, J. L. (2000). A yeast heterogeneous nuclear ribonucleoprotein complex associated with RNA polymerase II. *Genetics, 154*(2), 557-571.

Helmling, S., Zhelkovsky, A., & Moore, C. L. (2001). Fip1 regulates the activity of poly (A) polymerase through multiple interactions. *Molecular and cellular biology, 21*(6), 2026-2037.

Honorine, R., Mosrin-Huaman, C., Hervouet-Coste, N., Libri, D., & Rahmouni, A. R. (2011). Nuclear mRNA quality control in yeast is mediated by Nrd1 co-transcriptional recruitment, as revealed by the targeting of Rho-induced aberrant transcripts. *Nucleic acids research, 39*(7), 2809-2820.

Libri, D., Dower, K., Boulay, J., Thomsen, R., Rosbash, M., & Jensen, T. H. (2002). Interactions between mRNA export commitment, 3′-end quality control, and nuclear degradation. *Molecular and cellular biology, 22*(23), 8254-8266.

Minvielle-Sebastia, L., Winsor, B., Bonneaud, N., & Lacroute, F. (1991). Mutations in the yeast RNA14 and RNA15 genes result in an abnormal mRNA decay rate; sequence analysis reveals an RNA-binding domain in the RNA15 protein. *Molecular and cellular biology, 11*(6), 3075-3087.

Patel, D., & Butler, J. S. (1992). Conditional defect in mRNA 3'end processing caused by a mutation in the gene for poly (A) polymerase. *Molecular and cellular biology, 12*(7), 3297-3304.

Piruat, J. I., & Aguilera, A. (1998). A novel yeast gene, THO2, is involved in RNA pol II transcription and provides new evidence for transcriptional elongation-associated recombination. *The EMBO journal, 17*(16), 4859-4872.

Saguez, C., Schmid, M., Olesen, J. R., Ghazy, M. A. E.-H., Qu, X., Poulsen, M. B., . . . Jensen, T. H. (2008). Nuclear mRNA surveillance in THO/sub2 mutants is triggered by inefficient polyadenylation. *Molecular Cell, 31*(1), 91-103.

Steinmetz, E. J., & Brow, D. A. (1996). Repression of gene expression by an exogenous sequence element acting in concert with a heterogeneous nuclear ribonucleoprotein-like protein, Nrd1, and the putative helicase Sen1. *Molecular and cellular biology, 16*(12), 6993-7003.

Vasiljeva, L., & Buratowski, S. (2006). Nrd1 interacts with the nuclear exosome for 3′ processing of RNA polymerase II transcripts. *Molecular Cell, 21*(2), 239-248.
