# Supplementary figures and images for: Tho2‐mediated escort of Nrd1 regulates the expression of aging‐related genes
Source: Aging Cell. 2024 May 20;23(8):e14203. doi: 10.1111/acel.14203 (PMC11320360; doi:10.1111/acel.14203)

**Figure S1.**

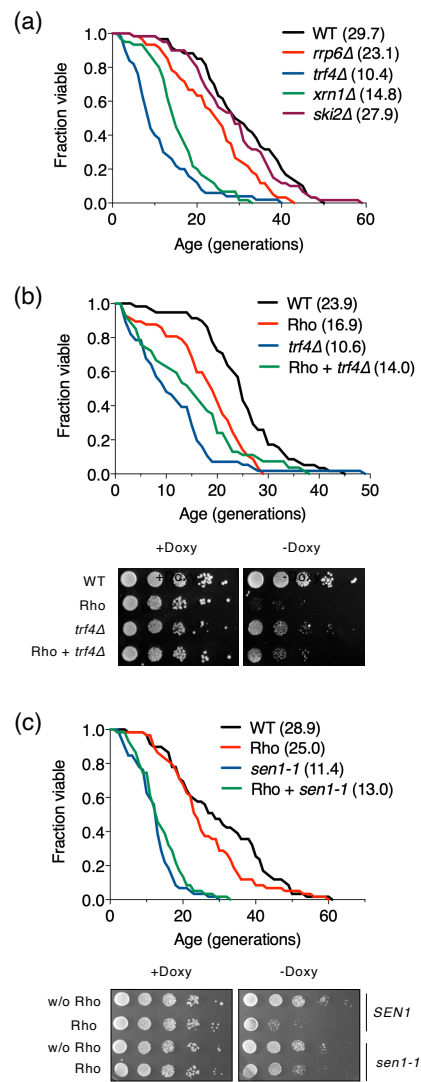

**Figure S2.**

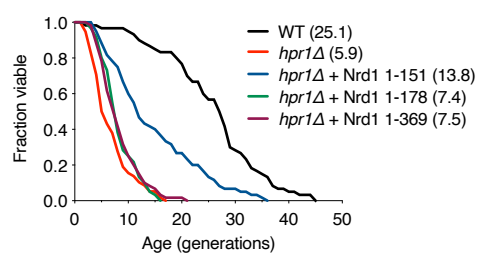

Figure S3.

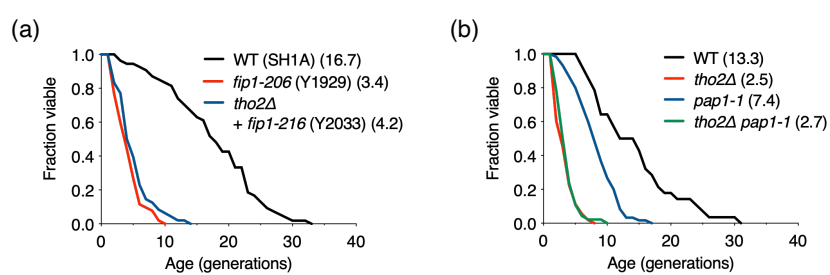

Supplement: Supplementary file 2 — FIGURE S1. Δ Figure S2. Δ Figure S3. [file ACEL-23-e14203-s001.pdf]
